# Identification of Novel Genomic Regions for Bacterial Leaf Pustule (BLP) Resistance in Soybean (*Glycine max* L.) via Integrating Linkage Mapping and Association Analysis

**DOI:** 10.3390/ijms23042113

**Published:** 2022-02-14

**Authors:** Fangzhou Zhao, Wei Cheng, Yanan Wang, Xuewen Gao, Debao Huang, Jiejie Kong, Augustine Antwi-Boasiako, Lingyi Zheng, Wenliang Yan, Fangguo Chang, Keke Kong, Ying-Yu Liao, Alejandra I. Huerta, Wusheng Liu, Mengchen Zhang, Tuanjie Zhao

**Affiliations:** 1 National Center for Soybean Improvement, Key Laboratory of Biology and Genetics and Breeding for Soybean, Ministry of Agriculture, State Key Laboratory of Crop Genetics and Germplasm Enhancement, Nanjing Agricultural University, Nanjing 210095, China; 2017201032@njau.edu.cn (F.Z.); chengwei20220101@outlook.com (W.C.); wangyanan02@baiyyy.com.cn (Y.W.); 2012094@njau.edu.cn (J.K.); bbbantwi@yahoo.com (A.A.-B.); 2018101147@njau.edu.cn (L.Z.); 2018201082@njau.edu.cn (W.Y.); 2017201054@njau.edu.cn (F.C.); 2017201030@njau.edu.cn (K.K.); 2 College of Plant Protection, Nanjing Agricultural University, Nanjing 210095, China; gaoxw@njau.edu.cn; 3 Department of Horticultural Science, North Carolina State University, Raleigh, NC 27607, USA; dhuang4@ncsu.edu (D.H.); wliu25@ncsu.edu (W.L.); 4 CSIR-Crops Research Institute, Kumasi AK420, Ghana; 5 Department of Entomology and Plant Pathology, North Carolina State University, Raleigh, NC 27607, USA; yliao22@ncsu.edu (Y.-Y.L.); ahuerta@ncsu.edu (A.I.H.); 6 National Soybean Improvement Center Shijiazhuang Sub-Center, North China Key Laboratory of Biology and Genetic Improvement of Soybean, Ministry of Agriculture, Laboratory of Crop Genetics and Breeding of Hebei, Cereal & Oil Crop Institute, Hebei Academy of Agricultural and Forestry Sciences, Shijiazhuang 050000, China

**Keywords:** linkage mapping, GWAS, qPCR, *Xanthornonas axonopodis* pv. *glycines*, bacterial leaf pustule, soybean

## Abstract

Bacterial leaf pustule (BLP), caused by *Xanthornonas axonopodis* pv. *glycines* (Xag), is a worldwide disease of soybean, particularly in warm and humid regions. To date, little is known about the underlying molecular mechanisms of BLP resistance. The only single recessive resistance gene *rxp* has not been functionally identified yet, even though the genotypes carrying the gene have been widely used for BLP resistance breeding. Using a linkage mapping in a recombinant inbred line (RIL) population against the Xag strain Chinese C5, we identified that quantitative trait locus (QTL) *qrxp–17–2* accounted for 74.33% of the total phenotypic variations. We also identified two minor QTLs, *qrxp–05–1* and *qrxp–17–1*, that accounted for 7.26% and 22.26% of the total phenotypic variations, respectively, for the first time. Using a genome-wide association study (GWAS) in 476 cultivars of a soybean breeding germplasm population, we identified a total of 38 quantitative trait nucleotides (QTNs) on chromosomes (Chr) 5, 7, 8, 9,15, 17, 19, and 20 under artificial infection with C5, and 34 QTNs on Chr 4, 5, 6, 9, 13, 16, 17, 18, and 20 under natural morbidity condition. Taken together, three QTLs and 11 stable QTNs were detected in both linkage mapping and GWAS analysis, and located in three genomic regions with the major genomic region containing *qrxp_17_2*. Real-time RT-PCR analysis of the relative expression levels of five potential candidate genes in the resistant soybean cultivar W82 following Xag treatment showed that of *Glyma.17G086300*, which is located in *qrxp–17–2*, significantly increased in W82 at 24 and 72 h post-inoculation (hpi) when compared to that in the susceptible cultivar Jack. These results indicate that *Glyma.17G086300* is a potential candidate gene for *rxp* and the QTLs and QTNs identified in this study will be useful for marker development for the breeding of Xag-resistant soybean cultivars.

## 1. Introduction

Bacterial leaf pustule (BLP), caused by the causal agent *Xanthomonas axonopodis* pv. *glycines* (Xag), is one of the most destructive foliar diseases in susceptible soybean cultivars. BLP causes a significant reduction in soybean yield and quality worldwide, especially in the soybean production areas in China [1], Korea [2], Thailand [3,4], the USA [5,6], and Benin [7], where seasonal high temperature and humidity favor Xag growth and development. Thus, BLP disease is more severe in South China, especially at Yangtze–Huai River Basins, in comparison with North China [1]. Recently, the incidence of BLP disease in South China has been rising along with global warming and the frequent occurrence of storms [1,8,9]. For example, the tropical storm ‘Rumbia’ caused an outbreak of BLP disease in the soybean growing area of Jianghuai River Basin in China in August 2018. In addition, it was reported that 88% of soybean growing in the fields in South Korea in 1998 were severely infected by Xag. Similarly, it accounted for 20.7–34.9% of soybean yield losses in Thailand in 1983 [2,10].

Xag infects soybean through natural openings and wounds on the abaxial surface of soybean leaves with the possibility to extend to stalks and petioles [5,11]. Typical BLP symptoms include small yellow-to-brown lesions with raised pustules in the lesion centers on infected soybean leaves, causing premature defoliation [5,11]. The pustule lesions caused by Xag can always be used as the infection sites by other microbial pathogens such as bacterium *Pseudomonas tabaci*, the causal agent of soybean wildfire disease [5]. Moreover, the BLP disease is easily misdiagnosed as Asian soybean rust caused by *Phakopsora pachyrhizi* [6], and the application of inappropriate pesticides could cause unnecessary economic burden to farmers.

To reduce economic losses in soybean production, disease resistance breeding is considered as the most cost-effective, efficient, and environmentally safe approach to achieve yield stability [12]. It was thought that a single major recessive resistance gene *rxp* confers BLP resistance while its dominant allele and associated genes regulate the degree of susceptibility [13,14]. The recessive *rxp* allele was originally determined in the soybean cv. CNS (P.I. 71,569), which is a highly BLP-resistant (near immune) cultivar and was developed in Nanjing, China [13]. To date, *rxp* has been widely used for the development of commercial BLP-resistant soybean cultivars worldwide [13,15]. In addition, *rxp* also confers resistance to soybean wildfire disease, which sometimes occurs with BLP disease [13].

The dominant *Rxp* allele, which confers susceptibility to the BLP disease, was originally found to be linked to the malate dehydrogenase (*Mdh*) locus with an about 16% recombination in a population of 650 F_2_ soybean plants developed from the crosses of Clark 63 and P.I. 437,477B [16]. Then, the *Rxp* locus was mapped to the genomic region between markers Satt372 and Satt014 on chromosome (Chr) 17 by using the hybrid offspring of resistant parent Coker 237 and susceptible parent P.I. 97,100, as well as the resistant parent Young and susceptible parent P.I. 416,937 [5].

QTL fine mapping located the recessive *rxp* in the 33-kb-long genomic fragment between markers SNUSSR17_9 and SNUSNP17_12 on Chr 17, which contains three genes, i.e., serine/threonine/tyrosine-protein kinase HT1 coding gene *Glyma17g09770*, membrane protein coding gene *Glyma17g09780*, and zinc finger protein coding gene *Glyma17g09790*; the latter two were predicted to be the key candidate resistance genes [2]. A GWAS study identified one significant SNP in each of *Glyma01g40560*, *Glyma01g40590*, *Glyma11g20310*, and *Glyma17g09801* that was associated with BLP resistance [17]. An RNA-Seq analysis identified the differentially expressed genes (DEGs) between resistant and susceptible soybean varieties under the infection of Xag strain 8ra at 0, 6, and 12 h post-inoculation (hpi) [18]. Interestingly, no significant difference was detected in the relative expression levels of *Glyma17g09780* and *Glyma17g09790* between the resistant and susceptible soybean varieties at 0, 6, and 12 hpi [18]. Several minor QTLs conferring resistance to various Xag isolates were also identified on Chr 18, 05, 04, 13, 19, and 10 in the recombinant inbred lines (RILs) derived from a cross between susceptible parent ‘Suwonl57’ and resistance parent ‘Danbaekkong’ using 76 SSR markers [14]. Furthermore, a novel QTL/gene responsible for BLP resistance was located at 21.5 cM away from the SSR marker Sat_108 on Chr 10 in P.I. 96,188, a soybean variety that only exhibits pustules without chlorotic haloes following Xag infection [19]. In addition, a few disease resistance genes were reported to be involved in BLP resistance in soybean. For example, the soybean *LATERAL ORGAN BOUNDARY1* (*GmLob1*; *Glyma.05g040500*) was predicted to be targeted by the virulence factor (tal2b) of Xag as its homolog *CsLOB1* in *Citrus sinensis* has been reported as a resistance gene to citrus canker disease caused by *X. citri* subsp. *Citri* (Xcc) [20,21]. A soybean lesion-mimic mutant *NT302* containing a defective hydroperoxide lyase (*HPL*) gene *Glyma.12g191400* (*GmHPL*) exhibited high susceptibility to Xag strain C5, indicating *GmHPL*’s role in Xag resistance [22].

In the present study, we assessed the BLP resistance level in a soybean RIL population and an association panel of soybean breeding lines in multiple environments. Then, we applied linkage mapping and GWAS for the identification of the BLP resistance QTLs and quantitative trait nucleotides (QTNs) in the soybean genome, leading to the identification of novel QTLs/QTNs. Our linkage mapping and GWAS revealed that BLP resistance is governed by one single major QTL *qrxp–17–2* and two minor QTLs *qrxp–05–1* and *qrxp–17–1* that were identified for the first time in the present study. The relative expression of five potential candidate genes in the resistant soybean cultivar W82 and susceptible cultivar Jack following Xag treatments showed that *Glyma.17G086300*, which is located in *qrxp–17–2*, is a candidate gene for *rxp*.

## 2. Results

### 2.1. The BLP Resistance of the Soybean RIL and Association Panel Lines and Their Relationship with Flowering Time

**Figure 1:**
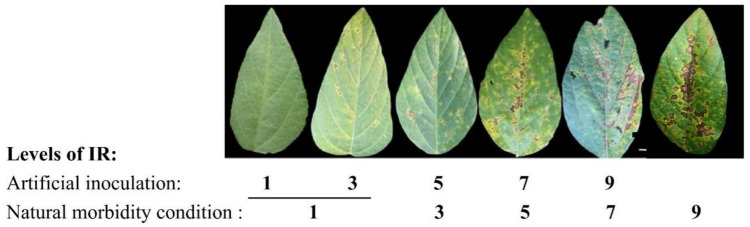
Bacterial leaf pustule disease symptoms scaled by the disease spot numbers. The BLP resistance performance of soybean plants were assessed as five levels, i.e., 1, 3, 5, 7, and 9. Scale bar of 1 cm was used.

BLP resistance was assessed in the soybean RIL population under the artificial inoculation condition and in the soybean association panel under both the artificial inoculation and natural morbidity conditions. The frequency distributions of the phenotypic data of soybean resistance to BLP disease exhibited a normal distribution pattern (Figure S1, see Supplementary Materials). Under the artificial inoculation condition, the mean infection responses (IRs) of the RIL in 2014–2015 and the association panel lines in 2014–2016 varied from 4.08 ± 2.71 to 5.65 ± 2.62 and from 2.76 ± 1.54 to 3.64 ± 1.76, respectively (Table 1). However, the mean IRs of the association panel lines under the national morbidity condition in 2018 varied from 2.54 ± 1.72 to 4.33 ± 1.88 (Table 1), indicating the severity of the BLP disease under the natural morbidity condition in 2018 (Figure 1). In addition, the BLP resistance levels in both populations showed a continuous variation from 1 to 9 (Tables S1–S3), demonstrating that the BLP resistance in these soybean lines is a quantitative trait and regulated by multiple genes. Analysis of variance (ANOVA) of the BLP resistance in each population under either condition (spray or the natural morbidity) indicated that the effects of genotype (G), environment (E), and genotype × environment (G × E) interactions were significant at the *p* < 0.001 level of significance (Table 1). The only exception came from the G × E interactions in the RIL lines under the spray condition, which were significant at the *p* < 0.05 level of significance. Moreover, the broad-sense heritability (*h*^2^) for BLP resistance in the two populations under either condition was 79.81% or greater (Table 1), indicating that the majority of phenotypic variation of BLP resistance in these soybean lines was attributed to effects of genotype (G).

Additionally, the flowering time of the soybean association panel was recorded under the natural morbidity condition in 2018 (Table S3) [23]. The correlation analysis showed a significant negative correlation at the *p* < 0.05 level (r = −0.29) between the BLP resistance level and flowering time, except the correlation was insignificant between the BLP resistance level and flowering time in the 2018DT-natural environment (Table S4).

### 2.2. Genetic Linkage Map Construction and Linkage Mapping in the Soybean RIL Population

A genetic map used for linkage mapping analysis was constructed in the soybean RILs. A total of 18.77 and 14.78 M reads and 1.58 and 1.24 Gbp bases of raw data were obtained for the two parents of the RIL population (Zhengyang and M8206), respectively. In addition, the mapped reads of two parents were 11.82 and 9.22 M and the mapped bases were 0.99 and 0.77 Gbp, respectively. The genome coverage of these two parents were 8.7 and 7.8%, respectively. For the RIL populations, a total of 66,677 SNPs were identified, and the average SNP density across the soybean 20 chromosomes was 6.85 SNPs per 100 kb (Table S5).

Using the composite interval mapping (CIM) method, we identified three QTLs, i.e., *qrxp_5_1*, *qrxp_17_1*, and *qrxp_17_2*, which were associated with BLP resistance in the RIL population in the 2014JP– and/or 2015JP–spray environments (Table 2). Among them, *qrxp_5_1* and *qrxp_17_1* were detected in the 2015JP–spray environment and located in the physical intervals of 1–1,169,356 bp on Chr 05 and 5,158,677–5,994,063 bp on Chr 17, respectively. The *qrxp_5_1* explained for 7.26% of phenotypic variation of BLP resistance with a negative effect, while *qrxp_17_1* accounted for 22.26% of the phenotypic variation of BLP resistance with a positive effect (Table 2). The *qrxp_17_2* was detected in both environments and located in the physical interval of 6,777,393–6,883,854 bp on Chr 17, explaining for 74.33% of phenotypic variance in the 2014JP–spray environment, and located in 6,293,843–6,883,854 bp on Chr 17, accounting for 34.68% of the phenotypic variance in the 2015JP–spray environment (Table 2). The *qrxp_17_2* showed a positive effect to BLP resistance and was considered as the most stable QTL associated with BLP resistance in soybean (Table 2).

### 2.3. Population Structure and Linkage Disequilibrium (LD) Analyses in the Soybean Association Panel

**Figure 2:**
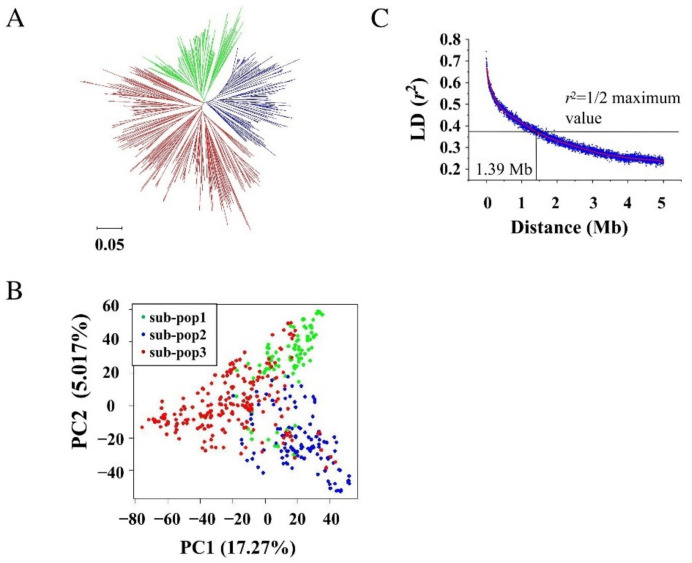
Population structure (**A**), principal component analysis (PCA; **B**), and linkage disequilibrium (LD) decay (**C**) of the 476 lines of the soybean association panel according to the high-density 61,166 soybean array. The LD parameters, viz., “*D*’” and “r^2^” were calculated. The LD decay rate of the population was measured as the chromosomal distance when the average r^2^ decreased to be half of its maximum value.

The 476 lines of the soybean association panel were genotyped using a high-density soybean array that consisted of 61,166 high-quality SNPs [24]. Clustering analysis using the neighbor joining method grouped all these lines into three groups (Figure 2A). The principal component analysis (PCA) also grouped them into three groups (Figure 2B). The first and second PCs explained for 17.27% and 5.02% of the total variance, respectively. The results of clustering analysis and PCA showed that the soybean lines derived from the same geographical locations and the same parental materials were generally grouped into the same subpopulations. The LD analysis showed that the r^2^ decreased gradually as the physical distance increased, and the LD decay distance was estimated at 1.39 Mb, where r^2^ dropped to being half of its maximum value of 0.74428 (Figure 2C).

### 2.4. Association Mapping in the Soybean Association Panel

**Figure 3:**
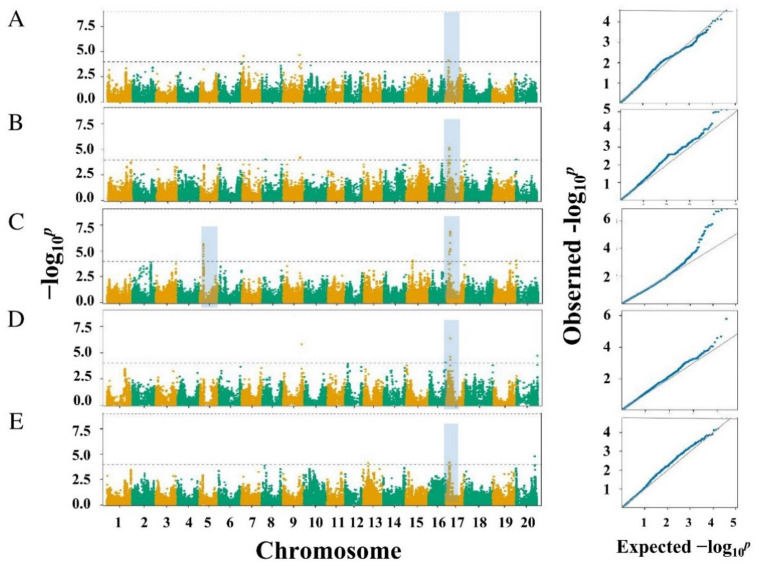
Manhattan and quantile–quantile (Q–Q) plots of genome-wide association study (GWAS) for BLP resistance in 476 soybean accessions in Jiangpu 2014 (**A**), 2015 (**B**), and 2016 (**C**) under artificial inoculation with C5 strain and in Dangtu (**D**) and Jiangpu (**E**) under natural morbidity condition. The *y*-axis indicates −log_10_ of *p*-values with significant association at 4.0 (dotted line).

BLP resistance in the association panel was evaluated under the artificial inoculation condition by using Xag C5 in Jiangpu from 2014 to 2016. The GWAS analysis detected 38 QTNs that were significantly associated with BLP resistance under the recommended threshold of −log10(*p*) ≥ 4 and distributed on eight chromosomes (Figure 3; Table S6). These QTNs explained for 3.26–6.04% of the phenotypic variation (Table S6). Among these QTNs, Gm17_7603802 and Gm17_7603992 on Chr 17 explained for the highest amount of phenotypic variation, i.e., 6.04%. A total of eight QTNs, i.e., Gm09_36501019, Gm17_7603802, Gm17_7603992, Gm17_7712768, Gm17_7721556, Gm17_7736150, Gm17_7754016, and Gm17_7754048, were identified in at least two environments (Table S6).

BLP resistance of the association panel was also evaluated under the natural morbidity condition in Jiangpu and Dangtu in 2018. The GWAS analysis revealed that 34 SNPs were associated with BLP assistance at the suggested threshold of −log10(*p*) ≥ 4 and distributed on nine chromosomes (Figure 3; Table S7). Among them, Gm17_7712768 explained for the phenotypic variation of 3.31–7.30% (Table S7). We found that 11 out of the 34 QTNs were co-localized with the QTNs identified in the same association panel under the artificial inoculation condition (Table 3). These 11 QTNs were Gm05_7667820, Gm05_7668047, Gm17_5628119, Gm17_5628133, Gm17_7603802, Gm17_7604008, Gm17_7712768, Gm17_7721556, Gm17_7736150, Gm17_7754016, and Gm17_7754048. A total of 13 and 11 stable QTNs associated with BLP resistance were identified in the association panel in ≥2 environments under both the artificial inoculation and natural morbidity conditions, respectively (Table 3; Tables S6 and S7). On the basis of the LD distance, we extended the significant associated region to cover 1.39 Mb upstream and downstream of the most significantly associated QTN positions (Table 3).

### 2.5. Co–Location Regions between Linkage Mapping and GWAS Analysis

**Figure 4:**
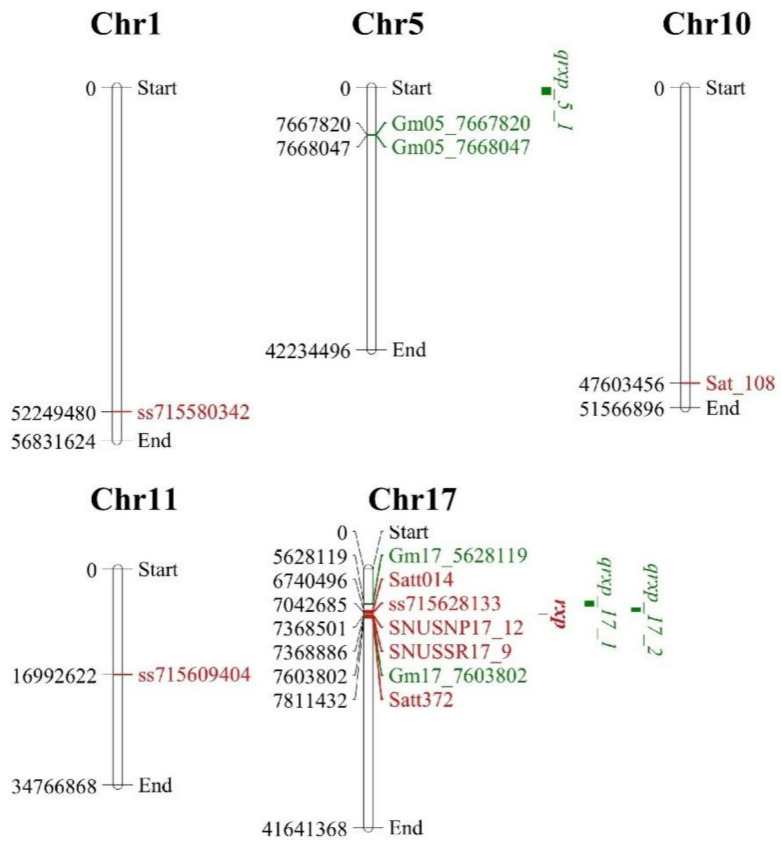
Distribution of the quantitative trait loci (QTLs) for BLP resistance and quantitative trait nucleotides (QTNs) related with BLP resistance in a physical map identified in previous and present studies. Red color indicates the QTLs and QTNs that have been previously reported; green color represents the QTLs and QTNs identified in the present study.

The three QTLs identified by linkage mapping and the 11 stable QTNs identified by GWAS were co-localized in three genomic regions (Table 2 and Table 3; Figure 4). QTL *qrxp_5_1* and two stable QTNs Gm05_7667820 and Gm05_7668047 were localized in genomic region 1, i.e., the 1–7,668,047 bp region on Chr 05. QTL *qrxp_17_1* and QTNs Gm17_5628119 and Gm17_5628133 were localized in genomic region 2, i.e., the 5,158,677–5,994,063 bp region on Chr 17. QTL *qrxp_17_2* was co-localized with eight stable QTNs in the 6,777,393–7,754,048 bp region on Chr 17 of genomic region 3. Thus, these genetic regions are closely linked to the causal effects of variations in the BLP resistance in soybean, making them robust regions for the identification of candidate genes.

### 2.6. Effects of the Most Significant Alleles in Three QTNs Individually or in Combination on BLP Resistance in Multiple Environments

**Figure 5:**
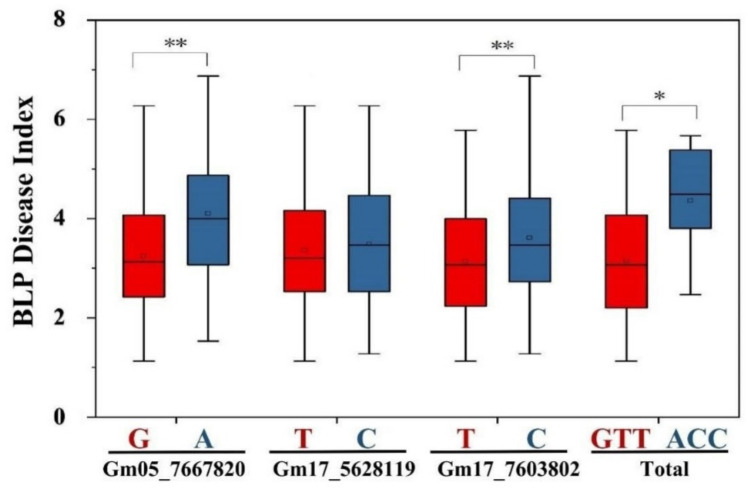
Effects of the alleles individually or in combination on BLP resistance over multiple environments. The bar graph shows the mean disease index. The *x*-axis indicates the QTNs. The red color indicates the resistant alleles, and the green color indicates the susceptible alleles. Genotypes with resistant alleles at each locus had the lowest mean disease index, while genotypes with susceptible alleles at each locus had the highest mean disease index. The *p*-values obtained from the *t*-test are indicated above each bar: * *p* ≤ 0.05, ** *p* ≤ 0.01.

Among the 11 stable QTNs identified by GWAS, Gm05_7667820, Gm17_5628119, and Gm17_7603802 explained for the highest phenotypic variation in the three co-localized genomic regions, respectively (Table 3). Thus, we assessed the effects of the most significant alleles G/A, T/C, and T/C in the QTNs Gm05_7667820, Gm17_5628119, and Gm17_7603802, respectively, on BLP resistance in multiple environments. The disease indexes of soybean cultivars containing Gm05_7667820 (G) and Gm17_7603802 (T) were significantly lower than that of soybean varieties containing Gm05_7667820 (A) and Gm17_7603802 (C) (Figure 5). Moreover, the disease indexes of the resistant soybean cultivars containing the three resistance alleles GTT were significantly lower than that containing the three susceptible alleles ACC (Figure 5). As a result, the cumulative effect of the three resistant alleles was able to increase the resistance for BLP disease in resistant soybean.

### 2.7. Prediction of Candidate BLP Resistance Gene in Soybean

**Figure 6:**
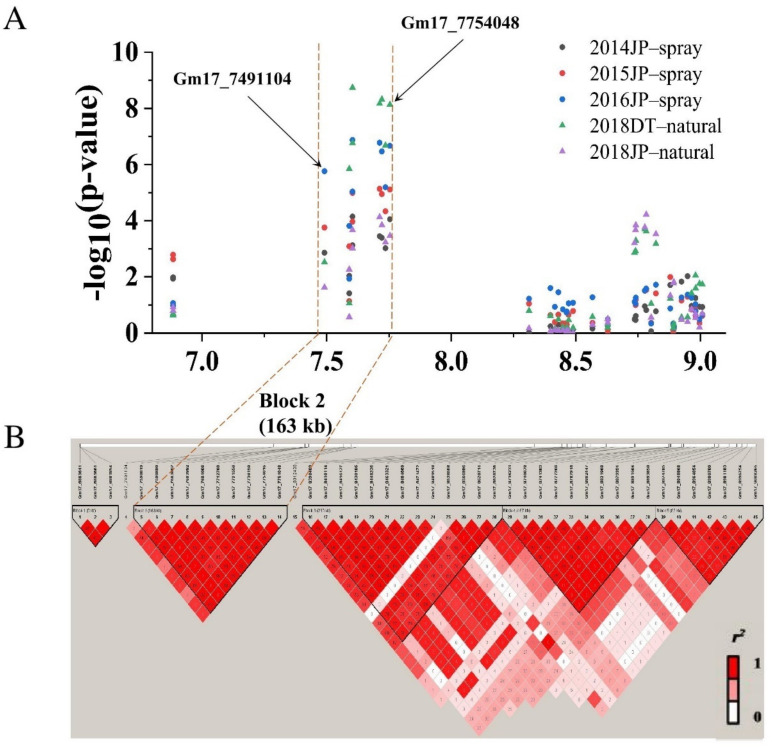
Haplotype–block analysis of the significant SNPs. (**A**) Association mapping results of the 6.88–9.01 Mb genomic region on chromosome 17. (**B**) Linkage disequilibrium (r^2^) values and haplotype blocks of the SNP markers observed in that region on chromosome 17. All the stable QTNs significantly associated with BLP resistance were located in the second LD block from 7.49 to 7.76 Mb containing 37 genes. Dark red, light red, and white color represent the strong, weak, and no LD between pairs of SNPs, respectively.

Among the three BLP resistance QTLs and the 11 stable BLP resistance QTNs co-localized in three genomic regions, the genomic region 3 covers the single major recessive resistance gene *rxp.* To further analyze the candidate genes of *rxp* in the genomic region 3, we downloaded all the 120 genes located in the region from the Soybase website (Table S8). Among them, 16 genes are located in *qrxp_17_2*. Haplotype analysis showed that the SNPs in this genomic region were clustered into five haplotype blocks (Figure 6). The stable QTNs associated with BLP resistance were clustered into the second haplotype block covering 21 genes located in the 170-kb-long genomic region from 7.59 to 7.76 Mb (Figure 6). The remaining 83 genes are located between *qrxp_17_2* and the second LD block (Table S8). Genes in the literature that meet one of the following criteria were predicted to be candidate BLP resistance genes: having been identified by gene mapping in soybean populations under Xag treatment, being responsive to the infection of Xag strains in soybean, and the paralogous genes responsive to the infection of *Xanthornonas* strains in other plant species. As a result, a total of four genes (i.e., *Glyma.17G086300*, *Glyma.17G090100*, *Glyma.17G090200*, and *Glyma.17G090400*) located between *qrxp_17_2* and the second LD block were identified to meet at least one of the three criteria (Table 4). Thus, these four genes together with *Glyma.05G040500* (the homolog of *Glyma.17G086300,* located near *qrxp_5_1*) were selected as the candidate BLP resistance genes for further analysis (Table 4).

### 2.8. Differential Expression Analysis of Candidate BLP Resistance Genes in Soybean

**Figure 7:**
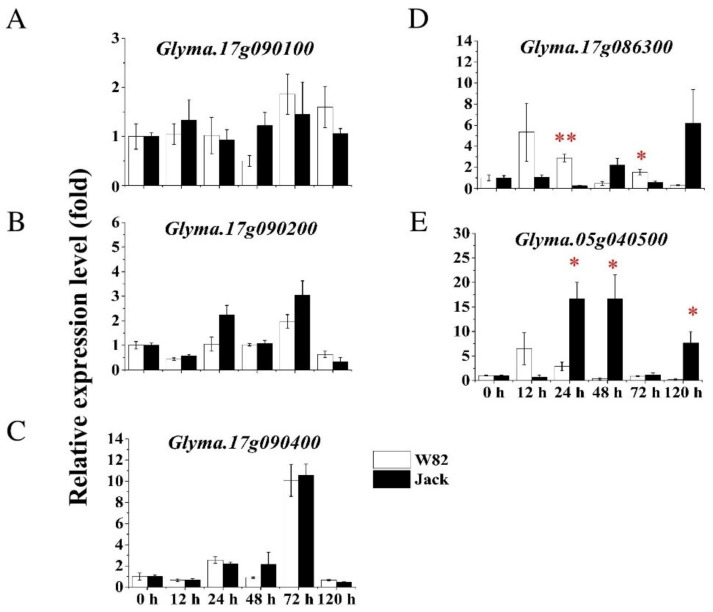
Relative expression levels of candidate BLP resistance genes *Glyma.17G090100* (**A**), *Glyma.17G090200* (**B**), *Glyma.17G090400* (**C**), *Glyma.17G086300* (**D**), and *Glyma.05G040500* (**E**) in BLP-resistant (W82) and -susceptible (Jack) soybean cultivars following Xag infection. The Xag strain EB08 was resuspended in 10 mM MgCl_2_ buffer to a final concentration of 1 × 10^8^ CFU/mL. Then, the bacterial suspension was injected on the leaves of W82 and Jack with 10 mM MgCl_2_ buffer being used as the mock treatment. The inoculated soybean leaves were sampled at 0, 12, 24, 48, 72, and 120 hpi, representing different stages of Xag infection. The relative expression levels were measured using qRT-PCR with the ATP-binding cassette transporter gene *Cons4* and an F-box protein family gene *Cons6* being used as the reference genes. The relative expression levels were calculated using the comparative threshold Pfaffl method. Values are the mean ± the standard error of three independent biological repetitions. Asterisks denote statistically significant difference (* *p* ≤ 0.05, ** *p* ≤ 0.01) between W82 and Jack by Student’s *t*-test.

In order to quantify the relative gene expression levels of each of the five candidate resistance genes in resistant and susceptible soybean cultivars following Xag (strain EB08) inoculation, we performed real-time RT-PCR (qPCR) in the leaf samples of cv. W82 (Xag resistant) and cv. Jack (Xag susceptible) at 0, 12, 24, 48, 72, and 120 hpi by using *Cons4* and *Cons6* as the internal control (or reference) genes (Tables S9 and S10) [25]. The average relative expression levels of *Glyma.17G090100*, *Glyma.17G090200*, and *Glyma.17G090400* in W82 showed no significant difference from that of Jack across the six time points after Xag EB08 treatments (Figure 7A–C). In comparison to Jack, however, the average relative expression levels of *Glyma.17G086300* was significantly increased in W82 at 24 and 72 hpi, while that of *Glyma.05G040500* was significantly decreased in W82 at 24, 48, and 120 hpi (Figure 7D,E).

## 3. Discussion

Soybean BLP is a worldwide disease and causes serious soybean yield reduction under high temperature and humidity. To date, only the soybean genotypes containing the unidentified recessive resistance gene *rxp* have been used in BLP resistance breeding, even though soybean BLP resistance is a quantitative trait that is regulated by multiple genes and affected by the interaction between genotypes and environment in the field. Although several BLP resistance QTLs have been identified previously, little is known about the underlying molecular mechanisms of BLP resistance in soybean. In the present study, we combined linkage mapping and GWAS to identify the BLP resistance QTLs and QTNs in soybean, leading to the identification of a potential candidate gene for *rxp*.

The linkage mapping and GWAS methods are frequently utilized to identify disease resistance QTLs. Linkage mapping is based on the co-segregation of genetic regions over the genome in bi-parental families [26,27]. A drawback of this method is that the construction of the RIL populations is very time-consuming and laborious. Another drawback of this method is that it is only able to detect allelic diversity between the bi-parents, and its resolution highly depends on the number of recombination events. Considering QTL intervals usually extend over several cM, this limited resolution makes it extremely challenging to identify the target genes. This challenge could be overcome by using GWAS, which studies nature populations composed of numerous varieties [28]. GWAS can be used to detect the loci with multiple alleles over a genome. It can also be used to narrow down the detected QTL regions [28]. This is why GWAS has been used to identify the target genes associated with traits in various plant species [23,29,30]. However, the effectiveness of GWAS was limited by factors such as the false positives, linkage disequilibrium, complex population structures, and the alleles with the substantial effects on trait phenotypes but low frequencies in the populations [31,32]. Thus, linkage mapping and association analysis can be used together to facilitate genetic architecture analysis and the identification of target genes underlying large QTLs [33,34].

Earlier studies have reported four QTLs/marker–trait association (MTA) genomic regions on Chr 01, 10, 11, and 17 in soybean, correspondingly, with the single recessive resistance gene *rxp* being located on Chr 17 [2,17,19]. In this study, we identified three genomic regions that contain three BLP resistance QTLs and 11 stable BLP resistance QTNs and were significantly associated with BLP resistance by combining linkage mapping and GWAS analysis (Figure 3; Table 2 and Table 3). Moreover, two new minor QTLs, *qrxp_5_1* (in genomic region 1) and *qrxp_17_1* (in genomic region 2), were identified on Chr 05 and 17, respectively, for the first time (Table 2). These two new QTLs were further confirmed by the co-localization of QTNs Gm05_7667820 and Gm05_7668047 with *qrxp_5_1* and QTNs Gm17_5628119 and Gm17_5628133 with *qrxp_17_1* (Table 2 and Table 3). Moreover, genomic region 3 containing *qrxp_17_2* and eight QTNs was located in the same genomic region as *rxp* (7.30 Mb) (Figure 4). We also evaluated the effects of the most significant SNPs Gm05_7667820, Gm17_5628119, and Gm17_7603802, which were distributed in the three genomic regions of soybean, on the improvement of BLP resistance. The average resistance of soybean lines containing all the three resistance alleles significantly increased when compared with that containing the three susceptible counterparts (Figure 5). Thus, BLP-resistant soybean cultivars can be developed through polymerization breeding. Furthermore, the QTLs and QTNs identified in this study can be used for marker-assisted selection of BLP-resistant soybean lines.

Using fine mapping and GWAS, Kim et al. [2] and Chang et al. [17] predicted *Glyma17g09780* (*Glyma.17G090100*), *Glyma17g09790* (*Glyma.17G090200*), and *Glyma.17g09801* (*Glyma.17G090400*) as the candidate genes for *rxp*. Among them, *Glyma.17G090100* is a casparian strip membrane domain protein (CASP) gene containing transmembrane regions and similar to that in Mildew Resistance Locus O (*MLO*) in barley, which confers powdery mildew resistance [35]. *Glyma.17G090200* is a C3H4-type zinc finger gene whose homolog in Arabidopsis, *AT3G47990*, encodes an E3 ubiquitin-protein ligase SIS3-like protein (SUGAR-INSENSITIVE 3), a positive regulator of sugar signaling during early seedling development, and is responsive to cabbage leaf curl virus (CaLCuV) infection [36,37]. *Glyma.17G090400* contains a significant SNP, bacterial pustule 1-g3, in the 5′-end untranslated region (5′-UTR) and encodes a calcium-dependent protein kinase 3 (CPK3), a key regulator of both pattern-triggered immunity (PTI) and effector-triggered immunity (ETI) in Arabidopsis [17,38]. However, all of these three genes are not differentially expressed genes (DEGs) between resistant and susceptible soybean varieties under the infection of Xag strain 8ra at 0, 6, and 12 h post-inoculation (hpi) [18].

Instead, we identified four candidate resistance genes for *rxp* in the present study. We further tested the relative expression levels of these four candidate genes together with *Glyma.05G040500* (the homolog of *Glyma.17G086300,* located near *qrxp_5_1*) in W82 (resistant) and Jack (susceptible) at 0, 12, 24, 48, 72, and 120 hpi under the infection of virulent Xag strain EB08. In order to overcome the dilution effect, we selected eight locations along the veins of each leaf for pathogen injection. In addition, we designed six sampling time points after treatment to prevent false negative results caused by insufficient sampling time points. According to the qPCR results, no significant difference was found between W82 and Jack at 12 hpi, which is consistent with the result of the reported RNA-Seq analysis [18]. However, we found that the expression of *Glyma.17G090400* increased 10 times in W82 and Jack under infection than mock treatment at 72 hpi. Interestingly, the relative expression levels of two Lob genes *Glyma.17G086300* and *Glyma.05G040500* showed a significant difference between W82 and Jack treated with EB08. When compared with that in Jack, the former significantly increased in relative expression levels in W82 at 24 and 72 hpi, while the latter significantly decreased in relative expression levels in W82 at 24, 48, and 120 hpi (Figure 7). *Glyma.17G086300* and its paralog *Glyma.05G040500* were predicted to be targeted by virulence factor tal2b, a transcription activator-like effector (TALE) of Xag [20]. *Xanthomonas* pathogens can secrete TALEs to activate multiple susceptibility genes in many plant spaces. For example, it can secrete TALE PthA to activate the expression of the canker susceptibility gene *CsLOB1* in citrus [21]. Taken together, *Glyma.17G086300* is a candidate gene for *rxp*.

## 4. Material and Methods

### 4.1. Plant Materials

A soybean RIL population and an association panel of soybean breeding lines were used in this study. The RIL population consisting of 126 F_2:9_ lines was developed by using the soybean cvs. Meng8206 and Zhengyang, which are resistant and susceptible to Xag strain C5, respectively [39], as the parents, and used for linkage mapping. These RIL lines along with the two parents were planted at Jiangpu Experimental Station, Nanjing, Jiangsu Province (latitude 33°03′ N; longitude 118°63′ E) in 2014 and 2015. The association panel containing 476 soybean breeding lines [24] was used for GWAS. These breeding lines were planted at Jiangpu Experimental Station in 2014, 2015, 2016, and 2018 and at Dangtu Experimental Station, Maanshan, Anhui Province (latitude 31°34′ N; longitude 118°29′ E) in 2018.

Seeds were sowed in a randomized complete blocks design (RCBD) with three replications with 1 × 0.25 m hill plots and 50 cm row spacing. Five soybean lines with five biological replicates per line were planted in each single row plot with 20 cm distance between accessions. Three seedlings of each line were kept and grown to maturity after seed germination. Field management was conducted under normal conditions.

### 4.2. Pathogen Inoculation

BLP resistance was assessed in these soybean plants under both artificial inoculation and natural morbidity conditions. The artificial inoculation was carried out by using a highly pathogenic Xag strain C5 [1] to evaluate BLP resistance in the soybean RIL and association panel lines at Jiangpu in 2014, 2015, and 2016, which were named as ‘2014JP–spray’, ‘2015JP–spray’, and ‘2016JP–spray’, respectively. The C5 strain was restreaked on nutrient agar (NA) medium (BBL; Becton Diskinson and Co., Cockeysville, MD, USA) from a 30% glycerol stock at −80 °C and incubated at 28 °C for 24–48 h [1]. Then, a single colony was grown overnight in the nutrient broth (NB) liquid medium (BBL; Becton Diskinson and Co. Ltd., Cockeysville, MD, USA) on a rotary shaker (220 rpm) at 28 °C. The bacterial culture was diluted to a final concentration of 3 × 10^8^ CFU/mL using sterilized distilled water and sprayed onto both sides of the soybean leaves at the early flowering stage with a high-pressure atomizer [40]. Ten days later, the inoculated plants were sprayed again with the same strain.

The natural morbidity condition in 2018 was used to evaluate BLP resistance in the association panel at Jiangpu and Dangtu Experimental Stations due to an outbreak of BLP disease in the soybean growing area of Jianghuai River Basin in China in August 2018, which resulted from the stormy weather brought by the tropical storm ‘Rumbia’. These inoculation environments were named as ‘2018JP–natural’ and ‘2018DT–natural’, respectively.

### 4.3. Disease Assessment

To evaluate the resistance of the soybean RIL and association panel lines under the artificial inoculation condition, we counted the disease spot numbers on the inoculated leaves of soybean plants at 14 days post-inoculation (DPI). The infection responses (IR) in the soybean genotypes under the artificial inoculation condition were grouped at five levels: Level 1, no apparent disease spots; Level 3, the existence of 5–20 localized disease spots; Level 5, the existence of 20–50 irregular spots; Level 7, the existence of >50 irregular disease spots that formed a small lesion covering 10–25% of leaf area; and Level 9, a large lesion covering more than 25% of leaf area (Figure 1).

When we considered the severity of the BLP disease under the natural morbidity condition in 2018, the five levels used to evaluate the susceptibility of soybean lines under the natural morbidity condition were Level 1, 0–20 disease spots; Level 3, 20–50 irregular disease spots; Level 5, >50 irregular disease spots that form a small lesion covering 10–25% of leaf area; Level 7, a large lesion covering 25–50% of leaf area; and Level 9, a large lesion covering more than 50% of leaf area (Figure 1).

Three biological replicates of each line were planted in a randomized complete block design with three blocks per environment. The average resistance levels of the three replicates per block in each environment were used for linkage mapping and association analysis.

### 4.4. Phenotypic Data Analysis

Descriptive statistics, such as mean, standard deviation (SD), maximum and minimum trait values, coefficients of variation (CV%), and skewness and kurtosis, in both soybean populations for BLP disease response were calculated using the origin pro 2018 software (Origin Lab, Northampton, MA, USA). The analysis of variance (ANOVA) for BLP disease resistance level of both soybean populations was performed to evaluate the effects of genotype (G), environment (E), and genotype × environment interaction (G × E) in the joint environments using the PROC GLM of SAS 9 (SAS Institute, 2010, Inc., Cary, NC, USA). Broad-sense heritability (*h*^2^) of BLP resistance of both soybean populations for combined environments was estimated as the following equation: *h*^2^ = σ^2^_g/_(σ^2^_g_ + σ^2^_ge/_n + σ^2^_e/_nr), where σ^2^_g_ is the genetic variance, σ^2^_ge_ is the genotype × environment interaction variance, σ^2^_e_ is the error variance, n is the number of environments, and r valid points is the number of replications within an environment [41].

### 4.5. Genetic Linkage Map Construction and Linkage Mapping in the Soybean RILs

RAD-seq (restriction-site-association DNA sequencing) [42] was used to genotype the 126 individuals of the RIL population and the two parents. Briefly, the genomic DNA was extracted from approximately 1 g of young leaves of individual plants of the RIL population and the two parents using the cetyltrimethylammonium bromide (CTAB) method [43]. The DNA fragments of 400~700 bp in length obtained by TaqI digestion were sequenced on Illumina HiSeq 2000 instrument following the standard protocol for MSG (multiplexed shotgun genotyping), and 90 mer paired-end reads were generated. The reads from RIL lines were aligned against the Williams 82 reference genome (Glyma.Wm82.a1.v1.1) [44] using the SOAP2 software (version 2.21) [45]. The SNP calling was performed using Real SFS software [46] on the basis of the Bayesian estimation. Finally, the high confidence SNPs were obtained by following the filtering criteria as follows: 50 <  depth <  2500, a probability of site mutation = 95%, and every SNP loci being separated by at least 5 bp. Bin maps were then constructed for the 126 RIL lines. Consecutive SNPs were examined with a slightly modified sliding-window approach (window size: 15 SNPs, step size: one SNP) [47]. Recombination breakpoints were determined as the window sledded along the chromosome. Windows with 11 or more SNPs from either parent and windows with fewer SNPs from a single parent were considered to be homozygous and heterozygous, respectively. The consecutive 30-kb-long intervals with the same genotype in each line were joined into a bin using a PERL script according to the breakpoint information [48]. Finally, the genetic map of bin markers was constructed for the RIL population using JoinMap 4.0 [49].

Composite interval mapping (CIM) model of WINQTLCartographer2.5 [50] was used to detect QTLs for BLP resistance in RILs in each environment. A bin map with 2600 bin markers was constructed, covering 2630.2 cM. The phenotypic variance explained by a single QTL was calculated by using WINQTLCartographer2.5 software with the PVE = (VG/VP) × 100% (PVE: phenotypic variation explanation; VG: genetic variance of QTL; VP: phenotypic variance). The walk speed was set at 1 cM, and the window size was set at 10 cM. A log likelihood of 2.5 was used as the threshold for the presence of QTLs [50]. The QTLs were named according to the normal nomenclature [49].

### 4.6. Genotyping, Population Structure, and LD Analysis in the Soybean Association Panel

Genotyping was conducted as described in Li et al. [26] and Karikari et al. [24]. Briefly, the genomic DNA of each plant in the association panel was extracted using the CTAB method [43] and genotyped by the restriction site-associated DNA sequencing (RAD-Seq) technology. After Taq I digestion, the DNA fragments of 400–600 bp in length were obtained and then sequenced using Illumina HiSeq 2000 instrument (Illumina, San Diego, CA, USA). After being filtered, the high-quality SNPs with a >5% minor allele frequency (MAF) were used for principal component analysis (PCA), kinship analysis, LD analysis, and GWAS analysis. The genotyping data are available in the NCBI database (PRJNA648781, https://www.ncbi.nlm.nih.gov/bioproject/?term=prjna648781, accessed on 20 May 2020) and the National Center for Soybean Improvement website (http://ncsi.njau.edu.cn/info/1149/2070.htm, accessed on 20 May 2020).

Population structure analysis including the PCA of whole-genome SNPs and the construction of the neighbor-joining tree for the 476 lines in the association panel was performed using the TASSEL software version 5.2 [51] as described in Karikari et al. [24].

The linkage disequilibrium (LD) between pairwise SNPs was analyzed by calculating the LD parameter value, i.e., the squared Pearson correlation coefficient (r^2^) using the RTM–GWAS V1.1 software [52]. A vcf format genotype file including the 60,315 high-quality filtered SNPs created by the TASSEL software was used as the input file in the RTM–GWAS V1.1 software. For LD analysis, the maximum inter-locus distance was set at 5 Mb, and the minimum r^2^ threshold was set at 0.05. The calculated r^2^ against the physical distance between pairs of SNP markers was visualized using the origin pro 2018 software (Origin Lab, Northampton, MA, USA) with scatter plot and polynomial fitting. The LD decay rate was the physical distance where the r^2^ dropped from its maximum value to the half [51].

### 4.7. Association Analysis and Haplotype Block Analysis

Association analysis was performed by running mixed linear model (MLM) including the principal component analysis matrix (PCA) and the kinship matrix (K), i.e., MLM (PCA+K) using the TASSEL software version 5.2 [51]. Manhattan and quantile–quantile plots (QQ–plot) were created by using the qqman R package to visualize the GWAS result [53]. The distribution of *p*-values and the significant loci associated with BLP resistance over the whole genome in the GWAS panel were shown in the Manhattan graph. The significant threshold −log10(*p*) = 4.0 was used to control the genome-wide type I error rate [53]. The SNPs detected in at least two environments were considered as being relatively stable. A haplotype block analysis was performed using the Haploview software (version 4.2) [54].

### 4.8. Gene Annotation and Candidate Gene Prediction

The names and physical locations of the identified genes were downloaded from the Soybase database (https://soybase.org, accessed on 20 May 2020). Gene annotations were conducted according to the NCBI database. All the genes that respond to Xag infection were considered as potential candidate genes.

### 4.9. qPCR

Total RNA extracted from leaf samples of soybean cvs. W82 (resistant) and Jack (susceptible) following the treatment of Xag strain EB08 at 0, 12, 24, 48, 72, and 120 hpi in Zhao et al. [25] was used for cDNA synthesis and qPCR analysis of the relative expression levels of the five candidate genes, i.e., *Glyma.17G090100*, *Glyma.17G090200*, *Glyma.17G090400*, *Glyma.17G086300*, and *Glyma.05G040500* in both cultivars. Three biological replicates were designed for each treatment, and three leaves of each biological replicate were infected as three technical replicates. Primer design and stepwise optimization of qPCR conditions including annealing temperatures, primer concentrations, and cDNA concentration ranges were conducted as described in Zhao et al. [25] so that R^2^ ≥ 0.99 and E = 100 ± 5% were achieved for the best primer pair for each gene. The relative expression levels were calculated using *Cons4* and *Cons6* as the two reference genes [25] and the Pfaffl method [55].

## Figures and Tables

**Table 1:** Descriptive statistics and ANOVA for BLP disease resistance in soybean RIL and association panel populations.

Population	Environment	Infection Response ^a^	Range	CV (%) ^b^	FG ^c^	FE ^d^	FG × E ^e^	*h*2 (%) ^f^
RIL	2014JP-spray	5.65 ± 2.62	1–9	46.53	7.97 **	116 **	1.62 *	79.81
	2015JP-spray	4.08 ± 2.71	1–9	66.49				
Association panel	2014JP-spray	3.64 ± 1.76	1–9	48.41	104.51 **	7.79 **	1.27 **	91.63
	2015JP-spray	3.62 ± 2.33	1–9	64.58				
	2016JP-spray	2.76 ± 1.54	1–8	55.98				
Association panel	2018JP-natural	4.33 ± 1.88	1–9	43.39	1425.04 **	9.60 **	2.56 **	87.43
	2018DT-natural	2.54 ± 1.72	1–8	68.00				

^a^ Mean ± standard deviation; ^b^ CV, coefficient of variation; ^c^ FG, the F-value of genotype; ^d^ FE, the F-value of environment; ^e^ FG × E, the F-value of genotype × environment; ^f^ *h*^2^, heritability. * Significance at *p* ≤ 0.005; ** significance at *p* ≤ 0.001.

**Table 2:** BLP-resistant QTLs identified by linkage mapping in the soybean RIL population under the artificial inoculation condition.

QTL	Chromosome	Genetic Position (cM)	LOD	Additive	R^2^ (%) ^a^	Confidence Interval (cM)	Physical Position (bp)	Environment
*qrxp_5_1*	5	0.01	4.17	−0.74	7.26	0.00~0.50	1~1,169,356	2015JP-spray
*qrxp_17_1*	17	26.91	9.08	1.32	22.26	24.60~27.90	5,158,677~5,994,063	2015JP-spray
*qrxp_17_2*	17	34.81	33.01	2.29	74.33	33.50~36.60	6,777,393~6,883,854	2014JP-spray
		32.81	15.99	1.67	34.68	31.60~34.80	6,293,843~6,883,854	2015JP-spray

^a^ Phenotypic variance (%) explained by the peak markers.

**Table 3:** QTNs associated with BLP resistance identified by GWAS in the soybean association panel under artificial and natural morbidity conditions.

QTNs	Chromosome	Physical Position (bp)	−log_10_*p* ^a^	r^2^ (%) ^b^	Environment	Significant Associated Region
**Gm05_7667820**	5	7,667,820	4.06	3.30	2016JP-spray	6,277,820–9,057,820
			4.21	3.42	2018DT-natural	
**Gm05_7668047**	5	7,668,047	4.06	3.30	2016JP-spray	
			4.21	3.42	2018DT-natural	
Gm09_36501019	9	36,501,019	4.65	3.87	2014JP-spray	35,111,019–37,891,019
			4.24	3.48	2015JP-spray	
**Gm17_5628119**	17	5,628,119	4.64	3.86	2016JP-spray	4,238,119–7,018,119
			4.45	3.65	2018DT-natural	
**Gm17_5628133**	17	5,628,133	4.64	3.86	2016JP-spray	
			4.45	3.65	2018DT-natural	
**Gm17_7603802**	17	7,603,802	4.14	3.39	2014JP-spray	6,213,802–8,993,802
			4.99	4.20	2015JP-spray	
			6.87	6.04	2016JP-spray	
			8.74	7.88	2018DT-natural	
**Gm17_7604008**	17	7,604,008	5.04	4.24	2016JP-spray	
			6.76	5.89	2018DT-natural	
Gm17_7603992	17	7,603,992	4.14	3.39	2014JP-spray	
			4.99	4.20	2015JP-spray	
			6.87	6.04	2016JP-spray	
**Gm17_7712768**	17	7,712,768	5.13	4.35	2015JP-spray	
			6.77	5.94	2018DT-natural	
			8.17	7.30	2018JP-natural	
			4.13	3.31	2015JP-spray	
**Gm17_7721556**	17	7,721,556	4.95	4.17	2016JP-spray	
			6.46	5.64	2018DT-natural	
			8.32	7.45	2015JP-spray	
**Gm17_7736150**	17	7,736,150	4.33	3.58	2016JP-spray	
			5.19	4.39	2018DT-natural	
			6.68	5.81	2014JP-spray	
**Gm17_7754016**	17	7,754,016	4.05	3.30	2015JP-spray	
			5.11	4.32	2016JP-spray	
			6.66	5.83	2018DT-natural	
			8.14	7.26	2014JP-spray	
**Gm17_7754048**	17	7,754,048	4.05	3.30	2015JP-spray	
			5.11	4.32	2016JP-spray	
			6.66	5.83	2018DT-natural	
			8.14	7.26		

^a^ *p*, the statistical *p*-value for the significance of the odds ratio in the GWAS. ^b^ r^2^ (%), phenotypic variance (%) explained by the peak markers. Bold QTNs indicate that the markers can be located by GWAS under both artificial inoculation and natural morbidity conditions. The significant associated region was 1.39 Mb upstream and downstream of the most significantly associated QTN positions.

**Table 4:** Candidate resistance genes of *rxp* on chromosome 17 selected for further analysis.

Wm82.a2.1	Homolog in *A. thaliana*	Gene Name	Reference
*Glyma.17G086300*	*AT5G63090*	Lateral organ boundaries (*LOB*) domain-containing protein 25	Kladsuwan et al., 2017
*Glyma.17G090100*	*AT2G36330*	CASP-like protein 4A3	Kim et al., 2010; Chang et al., 2016
*Glyma.17G090200*	*AT3G47990*	E3 ubiquitin-protein ligase SIS3-like	Kim et al., 2010; Chang et al., 2016
*Glyma.17G090400*	*AT4G23650*	N.A.	Chang et al., 2016

## Data Availability

Not applicable.

## Supplementary Materials

The following supporting information can be downloaded at: https://www.mdpi.com/article/10.3390/ijms23042113/s1.

## Abbreviations

BLP	bacterial leaf pustule
Xag	*Xanthornonas axonopodis* pv. *glycines*
GWAS	genome-wide association study
QTLs/QTNs	quantitative trait loci/nucleotides
MAS	marker-assisted selection
Psg	*Pseudomonas syringae* pv. *Glycinea*
RIL	recombinant inbred line
YHSBLP	Yangtze–Huai soybean breeding germplasm population
NA plate	nutrient agar plate
NB	nutrient broth; MSG, multiplexed shotgun genotyping
RAD–seq	restriction site-associated DNA sequencing
SD	standard deviation
CV%	coefficient of variation
ANOVA	analysis of variance
MCIM	mixed composite interval mapping
Add	additive effects
PVE	phenotypic variance explanation
MAF	minor allele frequency
MDR	missing data ratio
PCA	principal component analysis
LD	linkage disequilibrium
MLM	mixed linear model
K	kinship control
Q–Qplot	quantile–quantile plots
hpi	hours post-inoculation
SD	standard deviation
MR	moderately resistant
NJZM–RIL	RIL population consisting of 289 F_2:9_ lines derived through single seed descent
SSD	single seed descent; the method from F_2_ generation of Zhengyanghuangdou x Meng8206 cross
HLP	Helix–loop–helix
qPCR	real-time quantitative reverse-transcription PCR
