# Supplementary material for: Identification of Novel Genomic Regions for Bacterial Leaf Pustule (BLP) Resistance in Soybean (Glycine max L.) via Integrating Linkage Mapping and Association Analysis
Source: Int J Mol Sci. 2022 Feb 14;23(4):2113. doi: 10.3390/ijms23042113 (PMC8876204; doi:10.3390/ijms23042113)
Supplement: Supplementary file 1 [file ijms-23-02113-s001.zip › ijms-1557345-supplementary.pdf]

Supplemental Information – Zhao et al. Identification of novel genomic regions for bacterial leaf pustule (BLP) resistance in soybean (*Glycine max* L.) via integrating linkage mapping and association analysis

Table S1. BLP resistance in the soybean RIL population following the artificial inoculation with Xag C5 strain

S. No.	Lines	2014JP –spray	2015JP –spray	S. No.	Lines	2014JP –spray	2015JP –spray	S. No.	Lines	2014JP –spray	2015JP –spray
1	RIL_1	5.00	1.00	15	RIL_15	7.33	8.00	29	RIL_29	8.00	6.00
2	RIL_2	9.00	4.00	16	RIL_16	4.00	1.00	30	RIL_30	9.00	9.00
3	RIL_3	9.00	7.00	17	RIL_17	4.00	1.00	31	RIL_31	7.33	3.00
4	RIL_4	7.67	2.00	18	RIL_18	9.00	6.00	32	RIL_32	8.00	8.00
5	RIL_5	7.00	4.33	19	RIL_19	6.11	3.00	33	RIL_33	7.00	9.00
6	RIL_6	7.00	5.11	20	RIL_20	7.00	1.00	34	RIL_34	9.00	8.00
7	RIL_7	2.00	4.56	21	RIL_21	4.00	2.00	35	RIL_35	8.00	4.33
8	RIL_8	1.33	3.00	22	RIL_22	9.00	8.00	36	RIL_36	8.00	+
9	RIL_9	2.00	2.33	23	RIL_23	2.00	1.00	37	RIL_37	9.00	4.33
10	RIL_10	8.00	7.00	24	RIL_24	8.00	1.00	38	RIL_38	7.00	9.00
11	RIL_11	1.67	3.00	25	RIL_25	1.00	1.00	39	RIL_39	3.00	1.00
12	RIL_12	3.00	1.00	26	RIL_26	7.00	2.00	40	RIL_40	9.00	5.00
13	RIL_13	8.11	6.22	27	RIL_27	2.67	1.00	41	RIL_41	2.00	1.00
14	RIL_14	1.00	1.00	28	RIL_28	1.00	1.00	42	RIL_42	3.00	2.00

Table S1. (Cont.)

S. No.	Lines	2014JP –spray	2015JP –spray	S. No.	Lines	2014JP –spray	2015JP –spray	S. No.	Lines	2014JP –spray	2015JP –spray
43	RIL_43	6.67	7.00	60	RIL_60	3.00	1.00	77	RIL_77	8.00	8.00
44	RIL_44	2.00	4.00	61	RIL_61	5.33	1.00	78	RIL_78	2.00	2.00
45	RIL_45	2.00	4.00	62	RIL_62	2.00	2.00	79	RIL_79	8.00	8.00
46	RIL_46	6.00	4.00	63	RIL_63	7.00	6.00	80	RIL_80	3.00	2.00
47	RIL_47	2.00	5.00	64	RIL_64	8.00	8.00	81	RIL_81	6.22	5.00
48	RIL_48	2.67	1.00	65	RIL_65	7.78	6.00	82	RIL_82	3.00	2.00
49	RIL_49	3.00	1.00	66	RIL_66	4.00	2.00	83	RIL_83	7.00	7.67
50	RIL_50	9.00	7.00	67	RIL_67	8.00	2.00	84	RIL_84	7.67	5.00
51	RIL_51	7.00	6.00	68	RIL_68	8.00	5.89	85	RIL_85	3.00	1.00
52	RIL_52	5.00	2.00	69	RIL_69	2.00	1.00	86	RIL_86	4.00	1.00
53	RIL_53	9.00	5.00	70	RIL_70	2.00	2.00	87	RIL_87	8.00	7.00
54	RIL_54	8.22	7.33	71	RIL_71	2.00	1.00	88	RIL_88	7.00	5.00
55	RIL_55	9.00	6.00	72	RIL_72	6.00	9.00	89	RIL_89	3.00	1.00
56	RIL_56	8.00	8.00	73	RIL_73	6.00	1.00	90	RIL_90	5.00	1.00
57	RIL_57	2.00	1.00	74	RIL_74	3.33	1.00	91	RIL_91	2.00	3.00
58	RIL_58	8.00	6.67	75	RIL_75	2.00	1.00	92	RIL_92	5.00	3.00
59	RIL_59	9.00	8.00	76	RIL_76	5.22	4.67	93	RIL_93	7.22	7.00

Table S1. (Cont.)

S. No.	Lines	2014JP –spray	2015JP –spray	S. No.	Lines	2014JP –spray	2015JP –spray	S. No.	Lines	2014JP –spray	2015JP –spray
94	RIL_94	7.11	3.00	105	RIL_105	7.00	1.00	116	RIL_116	6.00	2.67
95	RIL_95	9.00	5.67	106	RIL_106	8.00	6.00	117	RIL_117	4.00	2.00
96	RIL_96	6.67	5.00	107	RIL_107	9.00	7.00	118	RIL_118	5.11	5.67
97	RIL_97	8.00	2.00	108	RIL_108	5.00	1.00	119	RIL_119	1.00	1.00
98	RIL_98	9.00	7.00	109	RIL_109	3.00	1.00	120	RIL_120	7.22	1.00
99	RIL_99	6.67	4.67	110	RIL_110	6.00	8.00	121	RIL_121	7.00	8.33
100	RIL_100	7.00	7.00	111	RIL_111	9.00	4.00	122	RIL_122	3.00	2.00
101	RIL_101	2.00	1.00	112	RIL_112	5.00	7.67	123	RIL_123	8.00	7.00
102	RIL_102	8.00	9.00	113	RIL_113	9.00	4.00	124	RIL_124	3.00	3.22
103	RIL_103	9.00	6.00	114	RIL_114	5.00	4.33	125	RIL_125	6.67	6.00
104	RIL_104	8.00	5.00	115	RIL_115	3.00	1.00	126	RIL_126	1.00	1.00

Table S2. BLP resistance in the soybean association panel under the artificial inoculation with Xag C5 strain

S. No.	Lines	2014JP–spray	2015JP–spray	2016JP–spray
1	94-14//chu/zhou-2	6.00	7.22	7.00
2	94-14//chu/zhou	1.00	7.00	3.00
3	T119-1	3.00	8.00	5.67
4	tongzhoudou（cai5）	7.00	4.00	1.67
5	xin/tong-2	1.00	7.33	4.00
6	tongzhoudou/tong 4219-1 B2	9.00	7.00	3.00
7	tongzhoudou/4219-1 F2	5.00	7.67	4.33
8	tong/f（1）	4.00	7.56	4.33
9	msb×T·fs	4.00	1.00	5.00
10	T890840	6.00	6.00	7.00
11	T890844	7.00	5.00	1.00
12	tong 4219B2-4 B2	5.00	4.00	3.00
13	tong/ai-1	7.00	6.22	3.67
14	118×tieye	8.00	7.00	5.00
15	94-14×T790659-2	6.00	1.00	2.33
16	tong/si2（1）	6.00	5.33	2.00
17	tong/si2（2）	5.00	4.11	4.33
18	R19/tong（1）	7.00	3.00	2.33

Table S2. (Cont.)

S. No.	Lines	2014JP–spray	2015JP–spray	2016JP–spray
19	86-4/fengbaimu（1）	2.00	2.67	4.33
20	R19/tong（2）	3.00	3.22	3.00
21	118F6/zhou92029-2	4.00	9.00	5.67
22	liufeng/taiwan75（1）	3.00	1.00	1.67
23	liufeng	3.00	2.00	2.33
24	you4/5545-1	3.00	1.00	1.00
25	liufeng/taiwan75-2	5.00	4.00	1.67
26	94-16/IA2077	5.00	4.00	3.00
27	94-16/IA2077-5	2.00	2.00	1.00
28	b23[94-16/HD（1）	3.00	2.67	1.67
29	b23[94-16/HD（2）	6.00	1.00	1.00
30	b23[94-16/HD（3）	4.00	1.00	1.00
31	b23-1[94-16/HD	4.00	1.67	3.00
32	94-14	3.00	1.67	2.00
33	94-14/shanning111	5.00	6.00	2.33
34	94-16	3.00	1.00	1.00
35	118/jidou7（1）	2.00	4.67	4.33
36	118/jidou7-3（1）	4.00	3.33	3.00

Table S2. (Cont.)

S. No.	Lines	2014JP–spray	2015JP–spray	2016JP–spray
37	118/jidou7-1（1）	4.00	4.00	3.67
38	118/jidou7-3（2）	7.00	4.00	2.33
39	118/jidou7-2	5.00	4.00	2.33
40	118/jidou7（2）	4.00	4.00	4.33
41	118/OT94-47-3	1.00	4.00	3.00
42	94-14/NJ16-67	6.00	7.00	3.67
43	tong/tai（1）	2.00	9.00	6.00
44	tong/tai（2）	4.00	7.33	5.00
45	tong/tai（3）	3.00	7.00	3.00
46	tong/tai（4）	3.00	7.00	3.00
47	liufeng/USP-1	3.00	1.00	2.33
48	usp/118（1）	3.00	2.00	2.33
49	usp/118（2）	4.00	2.00	2.33
50	USP//tong/chu-4	5.00	2.00	1.67
51	chuxiu	3.00	2.00	2.33
52	liufeng/USP-4	3.00	3.22	3.00
53	118/hei26（1）	4.00	1.00	5.00
54	88-48/T173（1）	4.00	2.00	2.33

Table S2. (Cont.)

S. No.	Lines	2014JP–spray	2015JP–spray	2016JP–spray
55	liufeng/USP（1）	5.00	2.67	3.00
56	ZS07301×shanning12	4.00	1.00	1.67
57	liufeng/USP-2	3.00	1.00	1.00
58	liufeng/USP（2）	5.00	3.11	1.67
59	ai/118-3（1）	2.00	5.67	1.67
60	ai/118	2.00	1.00	2.33
61	ai/118-1	6.00	3.00	2.33
62	94-14/2032-1（1）	3.00	4.00	3.00
63	94-14/2032-1（2）	5.00	3.00	5.00
64	118/hei26（2）	3.00	5.00	4.33
65	TY/NT-1（1）	7.00	5.11	2.00
66	94-16/ji31（1）	4.00	4.33	3.00
67	tong/hei26-1	4.00	5.00	7.00
68	tong/hei26	9.00	6.00	5.00
69	tong/hei8	4.00	9.00	6.33
70	tong/U95-5390	2.00	2.00	2.33
71	86-4/huaidou2hao(4)	4.00	3.00	2.00
72	118J7/yFR(1)	4.00	6.67	3.67

Table S2. (Cont.)

S. No.	Lines	2014JP–spray	2015JP–spray	2016JP–spray
73	118J7/yFR(8)	6.00	6.33	3.67
74	118J7/yFR(2)	5.00	5.00	3.00
75	118J7/yFR(3)	6.00	5.00	3.67
76	118J7/yFR(4)	4.00	5.00	3.00
77	118J7/yFR(5)	9.00	3.22	2.33
78	118J7/yFR(6)	6.00	3.00	3.00
79	118J7/yFR(7)	4.00	4.00	3.00
80	118/hefeng35-2（1）	6.00	3.67	5.00
81	118/hefeng35-2（2）	2.00	3.67	3.67
82	lu2/xiang//tong	3.00	5.00	3.67
83	tong/hefeng35-1	4.00	7.00	4.00
84	tong/hei8-2（1）	3.00	8.00	5.67
85	tong/hei8-2（2）	1.00	6.00	3.67
86	94-14/shanning 11	6.00	4.00	4.33
87	94-14/dongxin 202	4.00	7.00	4.33
88	94-16/N2899	3.00	1.00	1.00
89	94-16/N2899(1)	3.00	5.00	2.33
90	94-16×2899	4.00	3.00	4.33

Table S2. (Cont.)

S. No.	Lines	2014JP–spray	2015JP–spray	2016JP–spray
91	86-4/zhongzuo（1）	3.00	6.67	3.00
92	86-4/zhongzuo（2）	3.00	4.00	2.33
93	NIL91-1N/you4	1.00	5.33	3.00
94	NIL91-N/you4（1）	4.00	4.44	5.67
95	NIL91-N/you4（2）	6.00	4.00	2.33
96	jinda74/NN99-10	2.00	2.00	2.33
97	jinda74×99-10	4.00	1.00	2.33
98	86-4/sb（1）	6.00	7.00	3.00
99	7241/86-4（1）	3.00	1.00	1.67
100	1138/W82	2.00	1.00	1.00
101	TP5×1138-2-1	3.00	3.00	1.00
102	sp//zi/you4///sp	1.00	6.67	4.33
103	dongxin3hao	4.00	1.00	1.67
104	sb/jin74//9910	3.00	6.33	3.67
105	you/sb-1	1.00	5.56	2.33
106	you4/sb	2.00	7.00	3.00
107	shanxiye×agh-5	2.00	5.67	2.33
108	NG94-156	4.00	6.00	5.67

Table S2. (Cont.)

S. No.	Lines	2014JP–spray	2015JP–spray	2016JP–spray
109	88-48/4690（1）	5.00	3.00	5.00
110	94-16/863070-D36	4.00	2.00	1.67
111	94-14/zhongzuo97-1121	3.00	1.67	2.00
112	NG4690	1.00	9.00	5.00
113	nannong86--4	4.00	1.00	1.00
114	86-4/huaidou2hao(3)	5.00	2.00	3.67
115	t821058	4.00	1.00	1.67
116	t821096	1.00	1.00	1.00
117	T821069	5.00	1.00	1.00
118	T821060	4.00	2.00	1.00
119	t821082	1.00	1.00	1.67
120	86-4/huaidou2hao(1)	5.00	2.00	2.33
121	86-4/huaidou2hao(2)	4.00	1.00	3.00
122	86-4/Dsp-2	1.00	1.00	1.00
123	86-4/sp	1.00	1.00	1.67
124	86-4/sp2（1）	4.00	1.00	1.67
125	86-4/RLFI（1）	2.00	1.33	1.00
126	86-4/xiang//87-23（1）	2.00	1.00	1.00

Table S2. (Cont.)

S. No.	Lines	2014JP–spray	2015JP–spray	2016JP–spray
127	493-1/you4-2	4.00	3.00	1.00
128	86-4/RLFI（2）	1.00	1.00	1.67
129	86-4/sp-2	3.00	2.11	3.00
130	86-4/Sp（1）	4.00	1.00	1.67
131	86-4/Sp（2）	5.00	1.00	1.67
132	86-4/sp2（2）	2.00	2.00	1.00
133	86-4/tongzhoudou	1.00	8.00	4.33
134	7241/86-4（2）	3.00	1.00	1.67
135	zhe/118（1）	7.00	3.00	1.67
136	zhe/118-2	5.00	3.33	1.00
137	nannong87-17	2.00	3.00	4.33
138	nannong33（nannong513）	5.00	7.00	4.33
139	86-4/NH5	5.00	1.00	1.00
140	86-4/RLFI2	1.00	1.00	1.67
141	86-4/Rlfi2-2	1.00	2.00	1.67
142	86-4/7424F2-19（1）	6.00	6.00	5.00
143	86-4/7424F2-1	5.00	4.00	5.67
144	86-4/7424F2-19（2）	4.00	1.00	1.00

Table S2. (Cont.)

S. No.	Lines	2014JP–spray	2015JP–spray	2016JP–spray
145	86-4/you4（1）	6.00	1.00	1.67
146	88-48/AGH（1）	1.00	1.00	1.67
147	86-4/you4（2）	2.00	2.33	1.67
148	86-4/you4（3）	2.00	3.00	3.00
149	22225/86-4（1）	2.00	1.00	1.00
150	H5050/86-4（1）	3.00	4.00	1.00
151	H5050/86-4（2）	1.00	5.00	1.00
152	tong/hefeng35（1）	3.00	2.00	1.00
153	90L-2/tong	3.00	2.00	1.67
154	NN8-4/Forrest	1.00	1.00	1.67
155	88-48/Sp	4.00	1.00	1.67
156	86-4/nh5（1）	3.00	1.00	1.00
157	86-4/Vance-1（1）	1.00	1.00	1.00
158	86-4/Vance-2	4.00	1.00	1.00
159	86-4/Vance（1）	3.00	1.00	1.67
160	86-4/Vance（2）	2.00	1.00	1.67
161	86-4/Vance-1（2）	4.00	1.00	2.33
162	86-4/Vance（3）	2.00	1.00	2.33

Table S2. (Cont.)

S. No.	Lines	2014JP–spray	2015JP–spray	2016JP–spray
163	you96-5×Vance	4.00	1.00	2.33
164	73-932/zao18-1	3.00	3.11	2.33
165	73932×zao18	4.00	3.00	3.67
166	nannong73-935	7.00	5.00	3.00
167	73-932	7.00	4.00	3.00
168	73-935/6255（1）	2.00	1.33	1.67
169	73-932/6255-2	5.00	2.00	3.00
170	73-935/6255（2）	1.00	1.00	1.00
171	73-935/6255（3）	7.00	1.00	3.00
172	73-935×M9449	5.00	5.00	2.33
173	73-935×Z0102	4.00	5.22	2.00
174	nannong493-1	3.00	3.33	1.67
175	8831/you4//73935（1）	4.00	1.67	3.67
176	8831/you4//73935（2）	4.00	2.00	1.67
177	（Y4×88-31）×73-935	6.00	2.00	1.00
178	chu//nan/you-2	9.00	6.00	3.67
179	83-19	6.00	4.67	3.00
180	118/hefeng25	4.00	7.00	3.00

Table S2. (Cont.)

S. No.	Lines	2014JP–spray	2015JP–spray	2016JP–spray
181	you×（88-31×73-935）	2.00	1.00	1.67
182	you/88-31//73-935	1.00	4.56	3.00
183	youchun96-4/86-4（1）	5.00	1.00	3.00
184	youchun96-4/86-4（2）	3.00	2.00	2.33
185	youchun96-4/86-4（3）	3.00	1.67	1.00
186	HL05	4.00	1.00	1.00
187	86-4/fengbaimu（2）	3.00	1.00	1.67
188	86-4/fengbaimu（3）	9.00	5.00	1.00
189	you94-412/86-4	1.00	9.00	6.33
190	86-4/242(1)	5.00	3.00	2.33
191	86-4/242(2)	4.00	3.00	2.33
192	86-4/242(4)	6.00	3.00	4.33
193	86-4/242(5)	3.00	6.00	5.67
194	86-4/242(8)	3.00	5.00	3.67
195	86-4/242(6)	5.00	1.00	1.00
196	86-4/242(7)	4.00	6.00	5.00
197	86-4/242(10)	3.00	8.00	7.00
198	86-4/242(12)	3.00	3.00	3.00

Table S2. (Cont.)

S. No.	Lines	2014JP–spray	2015JP–spray	2016JP–spray
199	86-4/242(11)	4.00	7.00	5.67
200	cs1F/86-4（1）	7.00	1.67	1.67
201	cs1F/86-4（2）	3.00	3.22	1.67
202	cs1F/86-4（3）	2.00	3.67	3.00
203	cs1F/86-4（4）	5.00	3.00	3.00
204	cs1F/86-4（5）	3.00	3.67	1.00
205	7241/86-4（3）	3.00	3.22	5.00
206	86-4/sb（2）	6.00	1.00	1.00
207	7241/86-4（4）	2.00	7.00	3.00
208	73-935//90L-2/ye（1）	3.00	9.00	5.00
209	73-935//90L-2/ye（2）	4.00	9.00	5.00
210	73-935//90L-2/ye（3）	4.00	4.67	6.33
211	(S/y4)F2	4.00	4.00	3.00
212	aijiaozao	5.00	2.00	1.00
213	aijiaozao/26201（1）	4.00	1.00	1.67
214	aijiaozao/26201（2）	4.00	2.00	2.33
215	aijiaozao/26201（3）	3.00	1.00	2.33
216	youchun96-4/86-4（4）	3.00	2.33	1.00

Table S2. (Cont.)

S. No.	Lines	2014JP–spray	2015JP–spray	2016JP–spray
217	nannong502（nannong32）	2.00	2.11	1.67
218	nannong99-10	4.00	3.00	1.00
219	tongdou2006	5.00	2.67	1.67
220	86-4/xiang	7.00	5.00	6.33
221	CT-5	1.00	8.00	5.00
222	xiangshuidou	8.00	7.00	4.00
223	chudou1hao	4.00	3.00	1.67
224	suxian21	5.00	1.00	3.00
225	caidou6hao	5.00	2.00	1.00
226	412785-1×Y4	7.00	6.22	3.67
227	suxian19	1.00	1.00	2.00
228	88-31t	6.00	1.00	1.00
229	ai/118-3（2）	2.00	1.00	1.00
230	tongdou7hao	4.00	5.00	2.33
231	nannong88-31	6.00	4.11	1.67
232	88-31×（88-31×4985）	4.00	2.00	1.67
233	88-31×（88-31×4985）bao1	9.00	2.00	1.00
234	Y96-3×（Y4×88-31）（1）	4.00	1.00	1.00

Table S2. (Cont.)

S. No.	Lines	2014JP–spray	2015JP–spray	2016JP–spray
235	you94-412	3.00	5.33	2.33
236	USP90-2	4.00	5.00	4.33
237	qingpidou×N2899	4.00	6.00	3.67
238	CS1×fengdouFS	5.00	3.00	1.00
239	CS1×yu16	3.00	4.11	3.00
240	88-48×P1（1）	4.00	6.67	4.33
241	118/jidou4-3	5.00	1.00	1.00
242	118/jidou4-4	5.00	3.67	3.00
243	118J4/xiangyou	7.00	5.00	1.67
244	118/jidou7-1（2）	5.00	5.00	4.33
245	118/jidou7（3）	7.00	4.33	3.67
246	HD/94-16	4.00	3.89	3.67
247	V97-6490B/95C-13	4.00	1.00	1.00
248	sp//493-1/you（1）	1.00	1.00	4.33
249	88-48/T173（2）	4.00	8.00	4.00
250	Lar.r×36118	4.00	8.00	5.00
251	xin/tong	4.00	9.00	6.33
252	N/(xiang／88-48)（1）	1.00	8.00	6.33

Table S2. (Cont.)

S. No.	Lines	2014JP–spray	2015JP–spray	2016JP–spray
253	N/(xiang / 88-48)（2）	1.00	6.00	3.00
254	N/(xiang/88-48）	4.00	2.44	3.00
255	22033×69-3	2.00	5.00	6.33
256	88-48/SP	6.00	8.00	8.00
257	nannong88-48	3.00	1.00	1.67
258	88-48y	3.00	6.00	3.00
259	88-48/sb-f-p1-1	3.00	3.00	2.33
260	88-48×P1（2）	5.00	3.67	5.00
261	88-48/sb-f-P1（1）	3.00	5.67	6.33
262	88-48/sp-2	2.00	7.67	4.33
263	86-4/nh5（2）	1.00	3.67	2.33
264	86-4/NT-1（1）	5.00	2.00	3.00
265	88-48y/86-4y	1.00	7.00	3.67
266	N//xiang/88-48	5.00	4.67	1.67
267	88-48/p1 p1dan-1	1.00	4.00	3.00
268	NT-1	3.00	3.00	1.00
269	88-48/f（1）	3.00	8.00	5.67
270	88-48/f（2）	3.00	7.33	6.00

Table S2. (Cont.)

S. No.	Lines	2014JP–spray	2015JP–spray	2016JP–spray
271	88-48/nt-1--6	3.00	5.00	2.33
272	88-48/agh-2	2.00	4.00	3.00
273	Ty/88-48y-1	1.00	6.78	5.00
274	88-48//xin/tong	5.00	5.22	1.67
275	b94[88-48 yuan/94-16(1)	1.00	2.33	1.67
276	b94[88-48 yuan/94-16(2)	2.00	6.00	4.33
277	fendou51	5.00	6.00	6.00
278	kefeng1hao	1.00	7.00	7.00
279	fen33/94-5	3.00	4.00	4.33
280	86-4/21534（1）	1.00	2.00	1.00
281	86-4/N21534	5.00	4.67	3.00
282	jidou7hao	5.00	6.00	5.00
283	IA2077	4.00	2.00	2.33
284	ZS07259×hedou12	3.00	1.00	1.00
285	88-48/agh-1	3.00	1.00	1.67
286	L65-3366-C	2.00	2.00	3.00
287	fuyang434×nannongcaidou5hao	5.00	2.00	1.00
288	taixingheidou	4.00	6.00	2.33

Table S2. (Cont.)

S. No.	Lines	2014JP–spray	2015JP–spray	2016JP–spray
289	jilin31	4.00	8.00	4.33
290	you4/Graham	1.00	1.00	1.00
291	B295	5.00	6.33	3.00
292	Graham	3.00	1.00	1.00
293	22318×5	3.00	3.00	1.67
294	taiwan75	1.00	5.00	2.33
295	nannong39	5.00	3.00	1.00
296	73-935/6255（4）	2.00	2.83	1.67
297	su1dlm×agh	1.00	6.89	3.00
298	vance	1.00	2.00	2.33
299	zhongdou20×agh	3.00	1.00	5.00
300	tong/hefeng35（2）	1.00	1.00	2.33
301	shanxiye×agh-32	4.00	4.33	1.67
302	tong/95-5390	1.00	3.33	5.00
303	tong×95-5390	3.00	7.33	4.33
304	zhe3/118-2-2	4.00	5.00	4.33
305	zhe3/118-1-3	5.00	7.00	4.00
306	zhe/118（2）	3.00	6.00	4.33

Table S2. (Cont.)

S. No.	Lines	2014JP–spray	2015JP–spray	2016JP–spray
307	zhe3/118	2.00	5.67	4.33
308	zigongdongdou	3.00	9.00	6.33
309	zhechun3hao	5.00	8.22	5.00
310	you4/AGH	1.00	3.00	1.00
311	118/OT94-57-1(1)	3.00	3.00	2.33
312	118/hefeng35	2.00	4.67	3.67
313	94-16/ji31（2）	1.00	2.00	1.67
314	94-14//ji/dahuapi	3.00	3.00	1.00
315	liufeng/you4	3.00	2.00	1.67
316	94-16/p1（1）	3.00	1.00	1.67
317	94-16/p1（2）	1.00	1.00	1.67
318	94-16/y4(2)	6.00	1.00	2.33
319	94-16/p1（3）	1.00	4.00	1.67
320	94-16/p1（4）	3.00	2.67	2.33
321	han/you4（1）	5.00	5.78	5.00
322	han/you（1）	8.00	4.67	5.00
323	han/you（2）	2.00	1.00	1.00
324	han/you4（2）	1.00	2.00	2.33

Table S2. (Cont.)

S. No.	Lines	2014JP–spray	2015JP–spray	2016JP–spray
325	tong/tai（5）	4.00	1.00	4.33
326	hanyou	1.00	3.00	3.00
327	you/chuhun	5.00	1.67	1.00
328	heinong26	2.00	1.00	1.67
329	NH5	5.00	2.00	3.67
330	you4/ganyu	4.00	1.00	1.00
331	you4/5545（1）	5.00	1.00	1.00
332	you4/5545（2）	8.00	1.00	2.33
333	y4/5545	3.00	1.00	1.00
334	you4/88-31t-1	5.00	1.00	5.00
335	86-4/you4（4）	7.00	3.33	1.00
336	86-4/you4（5）	1.00	2.00	1.67
337	zhou92029-2	3.00	8.00	5.00
338	118/OT94-57-1(2)	3.00	5.00	3.67
339	118/OT94-57	4.00	3.00	3.00
340	88-48/87-17（1）	4.00	3.00	1.00
341	88-48/87-17（2）	2.00	4.33	1.00
342	88-48/87-17（3）	5.00	5.22	1.67

Table S2. (Cont.)

S. No.	Lines	2014JP–spray	2015JP–spray	2016JP–spray
343	88-48/87-17（4）	5.00	4.00	2.33
344	88-48/87-17（5）	5.00	4.00	1.00
345	88-48/RLFI	2.00	5.67	2.33
346	86-4/NT-1（2）	3.00	8.00	5.00
347	86-4/NT-1（3）	4.00	5.78	3.00
348	88-48/nanxiong//sb（1）	4.00	8.00	5.00
349	88-48/nanxiong//sb（2）	4.00	5.00	5.00
350	88-48/AGH（2）	2.00	6.67	3.67
351	88-48/21534	5.00	7.00	4.33
352	86-4/tongy	4.00	9.00	5.67
353	qihuang1hao×TF-2	1.00	1.00	1.00
354	（haixi×SP）×956251	4.00	3.22	1.67
355	88-48/nanxiong//sb（3）	6.00	8.00	7.00
356	88-48/nanxiong//sb（4）	3.00	9.00	7.67
357	nanxionghuangdou	5.00	8.33	7.00
358	hanchuanbayuebao	3.00	6.33	3.00
359	88-48/ hanchuanssd-3	4.00	5.00	3.00
360	tongshanbaopihuangdoujia	5.00	9.00	5.00

Table S2. (Cont.)

S. No.	Lines	2014JP–spray	2015JP–spray	2016JP–spray
361	HL03/94-14-3	5.00	5.00	3.67
362	HL03/94-14（1）	7.00	7.00	5.00
363	HL03/94-14（2）	4.00	3.00	1.00
364	7241/86-4（5）	4.00	6.00	4.33
365	sp//zi/you	3.00	1.00	2.33
366	88-48/lsp	1.00	4.00	5.00
367	88-48/4690（2）	6.00	1.00	1.67
368	D76-1609	5.00	1.00	3.00
369	22225/86-4（2）	3.00	2.00	1.67
370	87-23/86-4-2	4.00	1.00	1.00
371	87-23/86-4（1）	3.00	1.00	1.00
372	87-23/86-4（2）	5.00	1.00	1.00
373	86-4/xiang//87-23（2）	4.00	1.00	1.67
374	86-4/Vance（4）	1.00	3.00	3.67
375	tong/f（2）	3.00	1.00	1.67
376	tong/f1-1	3.00	1.67	3.00
377	tong/f（3）	7.00	1.00	2.33
378	tong/f1-2	4.00	1.00	1.00

Table S2. (Cont.)

S. No.	Lines	2014JP–spray	2015JP–spray	2016JP–spray
379	tong/f（4）	2.00	1.00	1.67
380	(86-4/xiang）/87-23	4.00	1.00	1.00
381	nannong1138-2	3.00	2.00	1.00
382	86-4/21534（2）	3.00	2.00	1.67
383	86-4/xiang//87-23（3）	2.00	1.00	1.00
384	86-4/xiang//87-23（4）	7.00	2.00	3.00
385	86-4/xiang//87-23（5）	3.00	7.00	5.00
386	86-4/xiang//87-23（6）	6.00	7.11	4.00
387	22369/88-48-2	5.00	1.00	1.00
388	22369/88-48（1）	6.00	1.00	1.00
389	22369/88-48（2）	7.00	1.67	1.67
390	you01-65×99-6（1）	5.00	2.00	1.67
391	996zao	4.00	1.00	1.67
392	nn99-6	4.00	1.00	1.67
393	TE99-6bu	4.00	1.00	2.33
394	you01-65×99-6（2）	2.00	2.00	1.67
395	you01-65×99-6（3）	5.00	1.00	1.67
396	Beeson	5.00	4.33	1.67

Table S2. (Cont.)

S. No.	Lines	2014JP–spray	2015JP–spray	2016JP–spray
397	xiangshuidou/you 4 （1）	5.00	3.00	2.33
398	88-48s/zheng0102	2.00	6.78	3.00
399	88-48×Z0102	5.00	4.00	2.33
400	zhe3/118-1	3.00	7.00	3.67
401	（nannongdahuangdou×JN03136） F1×wuxing2hao	3.00	5.78	2.33
402	N7241	3.00	1.00	1.00
403	guandou2hao	3.00	5.00	2.33
404	sp//493-1/you（2）	3.00	5.00	1.00
405	sp//493-1/y4	1.00	2.33	3.00
406	sp×（493-1×Y4）	4.00	3.00	1.00
407	sp//86-4/you（1）	5.00	5.33	3.67
408	sp//86-4/you（2）	5.00	6.00	2.33
409	R19S×200651	1.00	4.22	2.33
410	96-3//you4/8831（1）	2.00	1.00	1.00
411	Y96-3×（Y4×88-31）（2）	5.00	1.00	1.00
412	96-3//you4/8831（2）	1.00	1.00	1.00
413	493-1/you	3.00	1.00	1.67

Table S2. (Cont.)

S. No.	Lines	2014JP–spray	2015JP–spray	2016JP–spray
414	493-1/y4(gao)	2.00	7.89	5.00
415	493-1×Y4?	1.00	1.00	1.67
416	liufeng/taiwan75（2）	4.00	1.00	1.00
417	liufeng/taiwan75-1	3.00	3.00	3.00
418	you4/chu1（1）	2.00	3.00	1.67
419	you4/chu1（2）	3.00	2.33	1.67
420	nannong87-23	4.00	2.67	1.67
421	xiangshuidou/you 4 （2）	1.00	1.67	1.67
422	xiang/Y4	5.00	4.33	1.67
423	xiang/y4	1.00	1.00	1.67
424	86-4/you4-2	3.00	2.00	1.00
425	sp//86-4/you（3）	5.00	1.00	1.67
426	tong/HP1-1	3.00	5.22	3.67
427	you96-4	1.00	1.00	1.00
428	tong/HP	5.00	4.00	3.67
429	tong/DHP-1	5.00	2.00	2.33
430	tong/DHP	4.00	5.00	3.00
431	tong×DHP	1.00	5.22	3.00

Table S2. (Cont.)

S. No.	Lines	2014JP–spray	2015JP–spray	2016JP–spray
432	94-16/y4(1)	7.00	1.00	1.67
433	t06[tong/y4	3.00	5.00	3.67
434	zao1//liufeng/you4	1.00	1.00	1.67
435	zao1//liufeng/you)-2	3.00	1.00	3.00
436	94-16/p1（5）	3.00	4.25	2.33
437	94-16/p1（6）	5.00	2.00	1.67
438	94-16/p1（7）	3.00	2.67	1.00
439	sp//493-1/you（3）	4.00	2.00	1.00
440	you4/Graham-2	5.00	1.00	1.67
441	you4×L74-441	5.00	5.00	1.00
442	youchu4hao	3.00	4.00	3.00
443	493-1/y4	1.00	1.00	1.00
444	493-1/you4-1	5.00	2.00	1.67
445	xiang/y4(1)	5.00	1.00	1.67
446	zigong/you4（1）	3.00	1.00	1.67
447	zigong/you4（2）	3.00	2.00	1.00
448	zi/you-1	1.00	4.67	2.33
449	xinliuqing	1.00	1.00	1.67

Table S2. (Cont.)

S. No.	Lines	2014JP–spray	2015JP–spray	2016JP–spray
450	88-48/you	4.00	1.00	2.33
451	88-48/you4（1）	4.00	1.22	1.00
452	88-48/you4（2）	3.00	4.11	2.33
453	an×you	3.00	1.00	4.33
454	tong/you4（1）	3.00	3.00	3.00
455	tong/you4（2）	5.00	3.67	1.00
456	b23-2[94-16/HD	4.00	2.00	1.67
457	TY/NT-1（2）	1.00	5.00	2.33
458	TY/NT-1（3）	3.00	5.00	1.00
459	tong/dahuapi	5.00	3.00	3.67
460	88-48/sb-f-P1（2）	3.00	4.00	3.00
461	huaidou6hao×kexin3hao	5.00	7.00	3.67
462	IIfu×yu	3.00	5.00	3.67
463	you96-3	2.00	7.00	5.00
464	you4/5545（3）	3.00	5.00	2.33
465	Y-10-05	3.00	6.00	3.67
466	he95-1×peila	1.00	5.00	3.00
467	he95-1	4.00	7.22	5.00

Table S2. (Cont.)

S. No.	Lines	2014JP–spray	2015JP–spray	2016JP–spray
468	huaidou6hao×liaodou11	3.00	3.00	2.33
469	meng9024×peila	1.00	4.00	4.33
470	youbian30×yudou22（1）	1.00	6.22	4.33
471	Y-10-02	1.00	4.67	2.33
472	yudou22×peila	3.00	5.00	3.00
473	0	5.00	5.00	3.00
474	youbian30×yudou22（2）	1.00	5.00	3.00
475	tongdou8hao	1.00	6.33	3.00
476	JN03136×tai292	3.00	3.00	1.67

Table S3. BLP resistance and flowering time of the soybean association panel under the natural morbidity condition.

S. No.	Lines	2018JP–natural		2018DT–natural	
		DI	FT	DI	FT
1	94-14//chu/zhou-2	5.00	43	4.33	44
2	94-14//chu/zhou	2.33	57	5.00	57
3	T119-1	3.00	37	5.00	38
4	tongzhoudou（cai5）	5.00	41	3.67	44
5	xin/tong-2	5.00	42	7.00	44
6	tongzhoudou/tong 4219-1 B2	5.67	44	6.33	42
7	tongzhoudou/4219-1 F2	5.67	36	4.33	40
8	tong/f（1）	5.00	36	3.00	37
9	msb×T·fs	4.33	39	8.00	39
10	T890840	3.00	42	5.67	43
11	T890844	7.00	36	3.00	41
12	tong 4219B2-4 B2	5.67	44	5.00	46
13	tong/ai-1	7.00	36	3.00	38
14	118×tieye	5.00	41	5.00	41
15	94-14×T790659-2	5.00	43	1.00	45
16	tong/si2（1）	7.00	35	6.33	37
17	tong/si2（2）	3.67	36	4.33	40

Table S3. (Cont.)

S. No.	Lines	2018JP–natural		2018DT–natural	
		DI	FT	DI	FT
18	R19/tong（1）	5.67	36	1.00	38
19	86-4/fengbaimu（1）	1.67	44	4.33	46
20	R19/tong（2）	1.00	45	4.33	47
21	118F6/zhou92029-2	5.00	37	4.00	41
22	liufeng/taiwan75（1）	3.00	52	1.00	49
23	liufeng	3.67	42	1.00	44
24	you4/5545-1	9.00	39	1.00	40
25	liufeng/taiwan75-2	4.33	51	2.33	51
26	94-16/IA2077	7.00	40	1.00	43
27	94-16/IA2077-5	5.00	44	3.00	44
28	b23[94-16/HD（1）	4.33	37	1.00	41
29	b23[94-16/HD（2）	5.67	44	1.00	43
30	b23[94-16/HD（3）	5.67	39	1.00	40
31	b23-1[94-16/HD	9.00	36	1.00	37
32	94-14	3.00	57	3.00	57
33	94-14/shanning111	5.00	49	5.00	47
34	94-16	1.67	43	1.00	47

Table S3. (Cont.)

S. No.	Lines	2018JP–natural		2018DT–natural	
		DI	FT	DI	FT
35	118/jidou7（1）	3.00	42	3.00	49
36	118/jidou7-3（1）	5.67	36	5.00	47
37	118/jidou7-1（1）	5.00	41	3.67	41
38	118/jidou7-3（2）	7.00	51	3.00	52
39	118/jidou7-2	1.67	50	4.33	50
40	118/jidou7（2）	7.00	43	4.33	43
41	118/OT94-47-3	1.67	42	5.00	44
42	94-14/NJ16-67	1.67	43	1.67	40
43	tong/tai（1）	7.00	43	5.67	44
44	tong/tai（2）	3.00	36	5.67	38
45	tong/tai（3）	4.33	41	5.67	41
46	tong/tai（4）	7.00	49	2.33	51
47	liufeng/USP-1	3.00	44	1.00	47
48	usp/118（1）	7.00	32	2.33	34
49	usp/118（2）	5.00	38	1.00	42
50	USP//tong/chu-4	9.00	39	1.00	41
51	chuxiu	3.00	43	1.00	41

Table S3. (Cont.)

S. No.	Lines	2018JP–natural		2018DT–natural	
		DI	FT	DI	FT
52	liufeng/USP-4	4.33	45	1.00	47
53	118/hei26（1）	4.33	44	4.33	44
54	88-48/T173（1）	3.00	49	1.00	50
55	liufeng/USP（1）	5.67	44	2.33	46
56	ZS07301×shanning12	4.33	38	2.33	40
57	liufeng/USP-2	5.67	39	1.00	39
58	liufeng/USP（2）	2.33	42	2.33	46
59	ai/118-3（1）	1.00	41	7.00	44
60	ai/118	1.00	50	1.00	47
61	ai/118-1	5.00	38	3.67	37
62	94-14/2032-1（1）	3.00	47	3.00	45
63	94-14/2032-1（2）	4.33	37	3.67	36
64	118/hei26（2）	5.67	44	5.00	45
65	TY/NT-1（1）	5.00	41	3.00	46
66	94-16/ji31（1）	9.00	36	1.00	41
67	tong/hei26-1	5.00	39	5.00	43
68	tong/hei26	5.00	43	6.33	44

Table S3. (Cont.)

S. No.	Lines	2018JP–natural		2018DT–natural	
		DI	FT	DI	FT
69	tong/hei8	5.00	44	1.00	46
70	tong/U95-5390	1.00	39	1.67	44
71	86-4/huaidou2hao(4)	1.67	45	2.33	47
72	118J7/yFR(1)	5.67	56	4.33	57
73	118J7/yFR(8)	5.67	37	3.00	40
74	118J7/yFR(2)	4.33	37	2.33	41
75	118J7/yFR(3)	4.33	55	5.00	46
76	118J7/yFR(4)	5.00	36	3.67	40
77	118J7/yFR(5)	5.67	47	3.00	48
78	118J7/yFR(6)	5.67	38	2.33	37
79	118J7/yFR(7)	1.00	45	4.33	47
80	118/hefeng35-2（1）	5.00	38	4.33	40
81	118/hefeng35-2（2）	1.00	51	7.00	50
82	lu2/xiang//tong	4.33	35	2.33	37
83	tong/hefeng35-1	1.00	38	5.00	41
84	tong/hei8-2（1）	2.33	43	5.67	44
85	tong/hei8-2（2）	2.33	37	4.33	38

Table S3. (Cont.)

S. No.	Lines	2018JP–natural		2018DT–natural	
		DI	FT	DI	FT
86	94-14/shanning 11	5.67	40	2.33	47
87	94-14/dongxin 202	5.67	41	7.00	42
88	94-16/N2899	5.00	42	2.33	43
89	94-16/N2899(1)	4.33	39	2.33	40
90	94-16×2899	5.67	37	3.00	35
91	86-4/zhongzuo（1）	2.33	51	1.67	49
92	86-4/zhongzuo（2）	5.00	43	2.33	42
93	NIL91-1N/you4	1.00	44	2.33	46
94	NIL91-N/you4（1）	5.00	38	1.67	38
95	NIL91-N/you4（2）	5.67	45	4.33	46
96	jinda74/NN99-10	2.33	47	1.00	47
97	jinda74×99-10	4.33	50	2.33	50
98	86-4/sb（1）	7.00	62	2.33	59
99	7241/86-4（1）	4.33	41	1.00	44
100	1138/W82	2.33	39	1.00	39
101	TP5×1138-2-1	3.00	43	1.00	43
102	sp//zi/you4///sp	3.67	39	1.67	38

Table S3. (Cont.)

S. No.	Lines	2018JP–natural		2018DT–natural	
		DI	FT	DI	FT
103	dongxin3hao	4.33	39	1.00	38
104	sb/jin74//9910	1.00	43	5.67	47
105	you/sb-1	1.67	40	5.00	40
106	you4/sb	4.33	38	4.00	40
107	shanxiye×agh-5	3.00	49	4.33	50
108	NG94-156	5.00	44	5.00	46
109	88-48/4690（1）	5.00	47	3.00	48
110	94-16/863070-D36	5.00	40	3.00	42
111	94-14/zhongzuo97-1121	3.00	41	3.67	45
112	NG4690	3.00	47	5.00	46
113	nannong86--4	3.67	49	1.00	52
114	86-4/huaidou2hao(3)	3.67	53	1.00	52
115	t821058	3.67	41	1.00	41
116	t821096	2.33	51	1.00	51
117	T821069	5.00	46	1.00	48
118	T821060	5.00	37	1.00	37
119	t821082	1.00	53	1.00	51

Table S3. (Cont.)

S. No.	Lines	2018JP–natural		2018DT–natural	
		DI	FT	DI	FT
120	86-4/huaidou2hao(1)	5.00	42	1.00	45
121	86-4/huaidou2hao(2)	5.67	37	1.00	39
122	86-4/Dsp-2	3.00	37	1.00	42
123	86-4/sp	3.00	43	1.00	44
124	86-4/sp2（1）	5.00	43	1.00	45
125	86-4/RLFI（1）	7.00	38	1.00	43
126	86-4/xiang//87-23（1）	3.00	40	1.67	44
127	493-1/you4-2	1.00	51	1.00	43
128	86-4/RLFI（2）	1.67	42	1.00	45
129	86-4/sp-2	3.00	38	1.00	40
130	86-4/Sp（1）	5.67	42	1.00	42
131	86-4/Sp（2）	5.00	46	1.00	46
132	86-4/sp2（2）	3.00	41	1.00	43
133	86-4/tongzhoudou	1.00	46	3.67	45
134	7241/86-4（2）	4.33	55	1.00	55
135	zhe/118（1）	9.00	37	1.00	40
136	zhe/118-2	4.33	46	1.00	50

Table S3. (Cont.)

S. No.	Lines	2018JP–natural		2018DT–natural	
		DI	FT	DI	FT
137	nannong87-17	3.67	44	4.33	42
138	nannong33（nannong513）	5.67	42	3.00	43
139	86-4/NH5	1.00	47	1.00	53
140	86-4/RLFI2	6.00	37	1.00	38
141	86-4/Rlfi2-2	3.67	43	1.00	45
142	86-4/7424F2-19（1）	9.00	39	3.67	40
143	86-4/7424F2-1	5.00	42	1.67	44
144	86-4/7424F2-19（2）	1.00	45	1.00	47
145	86-4/you4（1）	3.00	37	1.00	40
146	88-48/AGH（1）	2.33	46	1.00	46
147	86-4/you4（2）	3.00	47	1.00	48
148	86-4/you4（3）	1.67	42	1.00	44
149	22225/86-4（1）	3.67	40	1.00	41
150	H5050/86-4（1）	5.00	40	1.00	46
151	H5050/86-4（2）	1.00	44	1.00	47
152	tong/hefeng35（1）	5.00	37	1.00	38
153	90L-2/tong	4.33	42	1.00	43

Table S3. (Cont.)

S. No.	Lines	2018JP–natural		2018DT–natural	
		DI	FT	DI	FT
154	NN8-4/Forrest	3.00	36	1.00	37
155	88-48/Sp	3.67	43	1.00	42
156	86-4/nh5（1）	4.33	44	1.00	48
157	86-4/Vance-1（1）	3.00	43	1.00	46
158	86-4/Vance-2	5.67	42	1.00	43
159	86-4/Vance（1）	3.00	47	1.00	48
160	86-4/Vance（2）	5.00	47	1.00	48
161	86-4/Vance-1（2）	2.33	46	3.67	46
162	86-4/Vance（3）	7.00	51	1.00	56
163	you96-5×Vance	3.67	43	1.00	44
164	73-932/zao18-1	1.00	44	3.00	49
165	73932×zao18	3.67	45	3.67	43
166	nannong73-935	5.00	51	1.00	54
167	73-932	5.67	43	2.33	44
168	73-935/6255（1）	3.00	47	1.00	48
169	73-932/6255-2	9.00	49	1.00	53
170	73-935/6255（2）	3.67	43	1.00	44

Table S3. (Cont.)

S. No.	Lines	2018JP–natural		2018DT–natural	
		DI	FT	DI	FT
171	73-935/6255（3）	5.00	37	1.00	40
172	73-935×M9449	5.00	46	1.00	50
173	73-935×Z0102	5.00	35	2.33	37
174	nannong493-1	5.67	29	2.33	40
175	8831/you4//73935（1）	5.00	39	1.67	41
176	8831/you4//73935（2）	4.33	44	3.00	44
177	（Y4×88-31）×73-935	3.00	55	1.00	56
178	chu//nan/you-2	5.67	54	1.67	51
179	83-19	3.67	50	3.67	45
180	118/hefeng25	3.00	55	4.33	46
181	you×（88-31×73-935）	1.00	44	1.00	46
182	you/88-31//73-935	5.00	36	5.67	42
183	youchun96-4/86-4（1）	5.00	35	1.00	35
184	youchun96-4/86-4（2）	1.67	43	3.67	46
185	youchun96-4/86-4（3）	3.67	44	1.00	45
186	HL05	4.33	43	1.67	50
187	86-4/fengbaimu（2）	3.00	52	1.00	49

Table S3. (Cont.)

S. No.	Lines	2018JP–natural		2018DT–natural	
		DI	FT	DI	FT
188	86-4/fengbaimu（3）	5.67	47	3.67	45
189	you94-412/86-4	5.67	47	7.00	51
190	86-4/242(1)	4.33	36	1.00	44
191	86-4/242(2)	3.00	38	1.00	41
192	86-4/242(4)	5.67	37	1.00	42
193	86-4/242(5)	5.00	37	1.00	38
194	86-4/242(8)	3.67	35	3.67	39
195	86-4/242(6)	5.67	42	1.67	41
196	86-4/242(7)	5.00	46	2.33	49
197	86-4/242(10)	5.00	49	2.33	51
198	86-4/242(12)	1.00	42	3.67	43
199	86-4/242(11)	2.33	39	3.67	38
200	cs1F/86-4（1）	5.00	43	1.00	44
201	cs1F/86-4（2）	3.00	37	4.33	40
202	cs1F/86-4（3）	3.67	42	1.00	44
203	cs1F/86-4（4）	3.00	37	1.00	41
204	cs1F/86-4（5）	5.67	37	1.67	39

Table S3. (Cont.)

S. No.	Lines	2018JP–natural		2018DT–natural	
		DI	FT	DI	FT
205	7241/86-4（3）	3.00	43	5.00	46
206	86-4/sb（2）	5.00	40	1.00	42
207	7241/86-4（4）	3.00	37	3.67	36
208	73-935//90L-2/ye（1）	1.00	45	5.67	44
209	73-935//90L-2/ye（2）	5.67	53	6.33	53
210	73-935//90L-2/ye（3）	5.67	37	5.67	39
211	(S/y4)F2	4.33	37	1.00	37
212	aijiaozao	5.00	36	1.00	39
213	aijiaozao/26201（1）	5.67	37	1.00	39
214	aijiaozao/26201（2）	3.67	40	1.00	41
215	aijiaozao/26201（3）	1.67	51	1.00	50
216	youchun96-4/86-4（4）	5.67	35	1.00	37
217	nannong502（nannong32）	3.00	38	1.67	41
218	nannong99-10	5.67	39	1.00	41
219	tongdou2006	3.67	39	1.00	40
220	86-4/xiang	5.00	42	2.33	44
221	CT-5	1.67	43	4.00	44

Table S3. (Cont.)

S. No.	Lines	2018JP–natural		2018DT–natural	
		DI	FT	DI	FT
222	xiangshuidou	4.33	42	5.00	44
223	chudou1hao	3.00	49	1.00	51
224	suxian21	5.67	40	1.00	39
225	caidou6hao	4.33	54	1.00	53
226	412785-1×Y4	5.00	51	5.67	49
227	suxian19	1.67	41	1.00	44
228	88-31t	7.00	36	1.00	39
229	ai/118-3（2）	2.33	51	1.67	48
230	tongdou7hao	3.67	51	1.00	50
231	nannong88-31	9.00	36	1.67	38
232	88-31×（88-31×4985）	1.67	44	1.00	42
233	88-31×（88-31×4985）bao1	5.00	45	1.00	45
234	Y96-3×（Y4×88-31）（1）	3.00	39	1.00	41
235	you94-412	1.00	43	5.67	44
236	USP90-2	3.67	38	3.67	43
237	qingpidou×N2899	1.67	42	3.00	46
238	CS1×fengdouFS	5.00	50	3.67	49

Table S3. (Cont.)

S. No.	Lines	2018JP–natural		2018DT–natural	
		DI	FT	DI	FT
239	CS1×yu16	3.00	48	5.00	43
240	88-48×P1（1）	3.00	56	2.33	57
241	118/jidou4-3	5.00	39	1.00	40
242	118/jidou4-4	5.00	38	2.33	40
243	118J4/xiangyou	3.00	40	3.00	41
244	118/jidou7-1（2）	3.67	39	3.00	40
245	118/jidou7（3）	9.00	39	5.67	44
246	HD/94-16	4.33	37	4.33	39
247	V97-6490B/95C-13	5.00	37	1.00	40
248	sp//493-1/you（1）	3.00	39	7.67	45
249	88-48/T173（2）	5.00	46	3.00	50
250	Lar.r×36118	4.00	45	3.67	45
251	xin/tong	3.00	40	4.33	42
252	N/(xiang / 88-48)（1）	1.00	43	3.67	48
253	N/(xiang / 88-48)（2）	1.00	39	5.00	43
254	N/(xiang/88-48）	4.33	39	3.00	44
255	22033×69-3	1.00	47	1.00	48

Table S3. (Cont.)

S. No.	Lines	2018JP–natural		2018DT–natural	
		DI	FT	DI	FT
256	88-48/SP	5.67	35	3.67	36
257	nannong88-48	2.33	44	1.00	47
258	88-48y	9.00	37	1.00	46
259	88-48/sb-f-p1-1	3.00	42	1.00	44
260	88-48×P1（2）	7.00	38	2.33	40
261	88-48/sb-f-P1（1）	2.33	49	2.33	46
262	88-48/sp-2	3.00	42	1.00	45
263	86-4/nh5（2）	3.67	42	5.00	44
264	86-4/NT-1（1）	7.00	43	2.33	44
265	88-48y/86-4y	3.00	41	4.33	44
266	N//xiang/88-48	5.67	38	1.67	37
267	88-48/p1 p1dan-1	3.67	42	1.00	44
268	NT-1	9.00	53	4.33	56
269	88-48/f（1）	1.00	42	1.67	43
270	88-48/f（2）	4.33	35	3.67	37
271	88-48/nt-1--6	5.00	43	6.33	44
272	88-48/agh-2	3.67	45	1.67	43

Table S3. (Cont.)

S. No.	Lines	2018JP–natural		2018DT–natural	
		DI	FT	DI	FT
273	Ty/88-48y-1	5.00	35	3.67	38
274	88-48//xin/tong	7.00	35	2.33	36
275	b94[88-48 yuan/94-16(1)	1.00	40	1.67	44
276	b94[88-48 yuan/94-16(2)	4.33	36	4.33	39
277	fendou51	7.00	43	5.67	46
278	kefeng1hao	7.00	36	6.33	38
279	fen33/94-5	4.33	34	2.33	37
280	86-4/21534（1）	3.67	43	1.00	44
281	86-4/N21534	3.00	43	1.00	43
282	jidou7hao	3.67	46	5.00	43
283	IA2077	5.67	41	1.00	39
284	ZS07259×hedou12	7.00	41	1.00	42
285	88-48/agh-1	3.00	37	3.00	41
286	L65-3366-C	3.67	38	2.33	42
287	fuyang434×nannongcaidou5hao	5.67	36	1.00	37
288	taixingheidou	5.00	37	5.00	38
289	jilin31	3.00	42	3.00	43

Table S3. (Cont.)

S. No.	Lines	2018JP–natural		2018DT–natural	
		DI	FT	DI	FT
290	you4/Graham	4.00	42	1.00	38
291	B295	5.00	44	3.00	47
292	Graham	4.33	41	1.00	41
293	22318×5	3.00	44	1.67	45
294	taiwan75	1.67	45	1.00	47
295	nannong39	3.67	42	1.00	41
296	73-935/6255（4）	1.67	46	1.00	48
297	su1dlm×agh	1.00	40	2.33	43
298	vance	1.67	36	1.00	39
299	zhongdou20×agh	7.00	40	1.67	40
300	tong/hefeng35（2）	4.33	37	5.00	42
301	shanxiye×agh-32	5.67	42	1.67	45
302	tong/95-5390	4.33	43	3.67	43
303	tong×95-5390	4.33	43	4.33	45
304	zhe3/118-2-2	3.67	43	3.67	46
305	zhe3/118-1-3	9.00	40	3.67	41
306	zhe/118（2）	5.67	39	3.67	41

Table S3. (Cont.)

S. No.	Lines	2018JP–natural		2018DT–natural	
		DI	FT	DI	FT
307	zhe3/118	4.33	39	5.00	40
308	zigongdongdou	5.00	45	1.00	46
309	zhechun3hao	5.00	40	5.67	40
310	you4/AGH	2.33	43	1.00	47
311	118/OT94-57-1(1)	7.00	37	5.00	39
312	118/hefeng35	2.33	47	2.33	47
313	94-16/ji31（2）	3.00	50	1.00	48
314	94-14//ji/dahuapi	5.67	43	2.33	47
315	liufeng/you4	5.00	39	3.67	43
316	94-16/p1（1）	5.67	42	1.00	45
317	94-16/p1（2）	5.00	51	4.33	51
318	94-16/y4(2)	5.00	43	1.00	45
319	94-16/p1（3）	1.67	41	3.67	48
320	94-16/p1（4）	3.00	42	1.00	46
321	han/you4（1）	9.00	40	5.00	41
322	han/you（1）	5.00	43	2.33	46
323	han/you（2）	3.00	43	1.00	47

Table S3. (Cont.)

S. No.	Lines	2018JP–natural		2018DT–natural	
		DI	FT	DI	FT
324	han/you4（2）	1.00	44	1.00	47
325	tong/tai（5）	3.00	45	1.00	47
326	hanyou	3.00	47	1.00	47
327	you/chuhun	3.67	47	1.00	48
328	heinong26	3.67	47	2.00	48
329	NH5	5.00	44	2.33	44
330	you4/ganyu	7.00	39	1.00	41
331	you4/5545（1）	9.00	38	1.00	40
332	you4/5545（2）	3.00	43	1.00	45
333	y4/5545	3.67	45	1.00	48
334	you4/88-31t-1	3.00	37	5.67	39
335	86-4/you4（4）	7.00	38	1.00	39
336	86-4/you4（5）	4.33	43	1.67	43
337	zhou92029-2	5.00	42	5.67	46
338	118/OT94-57-1(2)	2.33	45	2.33	47
339	118/OT94-57	7.00	45	5.00	47
340	88-48/87-17（1）	5.00	42	1.00	45

Table S3. (Cont.)

S. No.	Lines	2018JP–natural		2018DT–natural	
		DI	FT	DI	FT
341	88-48/87-17（2）	4.33	38	1.00	39
342	88-48/87-17（3）	4.33	43	1.00	45
343	88-48/87-17（4）	5.00	42	1.67	45
344	88-48/87-17（5）	5.00	45	1.00	50
345	88-48/RLFI	5.00	40	1.67	40
346	86-4/NT-1（2）	3.67	37	4.33	39
347	86-4/NT-1（3）	5.67	40	4.33	40
348	88-48/nanxiong//sb（1）	5.00	44	5.00	45
349	88-48/nanxiong//sb（2）	7.00	41	5.00	41
350	88-48/AGH（2）	2.33	42	3.00	44
351	88-48/21534	7.00	38	3.67	39
352	86-4/tongy	4.33	43	5.00	48
353	qihuang1hao×TF-2	3.67	45	1.00	48
354	（haixi×SP）×956251	5.67	44	1.00	47
355	88-48/nanxiong//sb（3）	7.00	39	6.33	40
356	88-48/nanxiong//sb（4）	3.00	45	5.00	47
357	nanxionghuangdou	9.00	39	4.00	41

Table S3. (Cont.)

S. No.	Lines	2018JP–natural		2018DT–natural	
		DI	FT	DI	FT
358	hanchuanbayuebao	5.67	43	3.00	46
359	88-48/ hanchuanssd-3	4.33	41	5.00	44
360	tongshanbaopihuangdoujia	5.00	37	5.00	36
361	HL03/94-14-3	5.00	51	3.67	52
362	HL03/94-14（1）	5.00	43	6.33	42
363	HL03/94-14（2）	4.33	37	3.00	39
364	7241/86-4（5）	5.67	38	1.67	42
365	sp//zi/you	5.67	41	1.00	39
366	88-48/lsp	2.33	43	5.00	44
367	88-48/4690（2）	7.00	40	1.00	42
368	D76-1609	5.00	46	1.00	49
369	22225/86-4（2）	4.33	44	1.00	46
370	87-23/86-4-2	7.00	37	1.00	40
371	87-23/86-4（1）	1.00	53	1.00	54
372	87-23/86-4（2）	7.00	37	1.00	39
373	86-4/xiang//87-23（2）	4.33	42	1.67	40
374	86-4/Vance（4）	1.00	50	1.00	50

Table S3. (Cont.)

S. No.	Lines	2018JP–natural		2018DT–natural	
		DI	FT	DI	FT
375	tong/f（2）	3.67	46	1.00	49
376	tong/f1-1	7.00	40	1.00	41
377	tong/f（3）	5.00	43	1.00	46
378	tong/f1-2	3.67	47	1.00	46
379	tong/f（4）	3.67	50	1.67	52
380	(86-4/xiang）/87-23	6.00	35	1.00	37
381	nannong1138-2	1.67	48	1.00	48
382	86-4/21534（2）	3.00	42	1.00	44
383	86-4/xiang//87-23（3）	1.67	45	2.33	45
384	86-4/xiang//87-23（4）	5.00	43	1.00	45
385	86-4/xiang//87-23（5）	1.67	40	5.00	41
386	86-4/xiang//87-23（6）	5.00	44	2.33	45
387	22369/88-48-2	5.67	43	1.00	46
388	22369/88-48（1）	7.00	45	1.00	45
389	22369/88-48（2）	5.00	46	1.00	47
390	you01-65×99-6（1）	9.00	45	1.00	47
391	996zao	3.00	43	1.67	46

Table S3. (Cont.)

S. No.	Lines	2018JP–natural		2018DT–natural	
		DI	FT	DI	FT
392	nn99-6	3.67	43	1.00	46
393	TE99-6bu	1.67	44	1.00	46
394	you01-65×99-6（2）	5.67	38	2.33	41
395	you01-65×99-6（3）	5.67	43	1.00	44
396	Beeson	5.67	39	2.33	40
397	xiangshuidou/you４（1）	5.00	37	1.00	43
398	88-48s/zheng0102	9.00	44	2.33	49
399	88-48×Z0102	3.67	43	4.33	45
400	zhe3/118-1	5.00	44	3.67	44
401	（nannongdahuangdou×JN03136）F1×wuxing2hao	9.00	41	5.00	44
402	N7241	3.33	43	1.00	45
403	guandou2hao	3.67	49	1.00	49
404	sp//493-1/you（2）	3.67	51	5.00	54
405	sp//493-1/y4	1.00	44	3.00	44
406	sp×（493-1×Y4）	3.00	53	1.67	55
407	sp//86-4/you（1）	5.00	37	1.00	36
408	sp//86-4/you（2）	5.67	47	1.67	50

Table S3. (Cont.)

S. No.	Lines	2018JP–natural		2018DT–natural	
		DI	FT	DI	FT
409	R19S×200651	3.00	53	2.33	54
410	96-3//you4/8831（1）	3.67	50	1.00	47
411	Y96-3×（Y4×88-31）（2）	5.00	41	1.00	43
412	96-3//you4/8831（2）	5.67	41	1.00	42
413	493-1/you	4.33	46	1.00	45
414	493-1/y4(gao)	3.67	43	5.00	44
415	493-1×Y4?	4.33	47	1.00	48
416	liufeng/taiwan75（2）	4.33	53	1.00	49
417	liufeng/taiwan75-1	4.33	42	1.67	43
418	you4/chu1（1）	5.00	56	1.67	52
419	you4/chu1（2）	3.00	41	1.00	41
420	nannong87-23	5.00	44	1.00	46
421	xiangshuidou/you 4 （2）	1.00	53	1.00	49
422	xiang/Y4	4.33	47	2.33	47
423	xiang/y4	3.67	43	1.67	45
424	86-4/you4-2	5.00	40	1.00	43
425	sp//86-4/you（3）	5.67	39	1.00	39

Table S3. (Cont.)

S. No.	Lines	2018JP–natural		2018DT–natural	
		DI	FT	DI	FT
426	tong/HP1-1	2.33	45	5.00	46
427	you96-4	2.33	43	5.67	47
428	tong/HP	2.33	43	4.33	42
429	tong/DHP-1	3.00	44	5.67	47
430	tong/DHP	5.67	37	4.33	37
431	tong×DHP	1.00	33	4.00	36
432	94-16/y4(1)	5.00	39	3.00	44
433	t06[tong/y4	5.67	39	3.67	40
434	zao1//liufeng/you4	1.00	36	1.00	38
435	zao1//liufeng/you)-2	5.67	41	3.67	42
436	94-16/p1（5）	7.00	37	1.67	38
437	94-16/p1（6）	5.67	37	1.00	41
438	94-16/p1（7）	5.00	33	1.00	42
439	sp//493-1/you（3）	4.33	38	2.33	39
440	you4/Graham-2	5.00	45	1.00	42
441	you4×L74-441	7.00	43	1.67	41
442	youchu4hao	3.67	39	1.00	39

Table S3. (Cont.)

S. No.	Lines	2018JP–natural		2018DT–natural	
		DI	FT	DI	FT
443	493-1/y4	3.67	38	1.00	37
444	493-1/you4-1	4.33	42	4.33	41
445	xiang/y4(1)	7.00	39	1.00	40
446	zigong/you4（1）	9.00	42	1.00	45
447	zigong/you4（2）	9.00	44	1.00	46
448	zi/you-1	2.33	55	1.67	48
449	xinliuqing	3.67	36	1.00	35
450	88-48/you	4.33	41	2.33	42
451	88-48/you4（1）	9.00	42	5.00	41
452	88-48/you4（2）	4.33	39	3.00	38
453	an×you	4.33	44	1.00	44
454	tong/you4（1）	1.00	49	1.00	47
455	tong/you4（2）	5.00	45	3.00	47
456	b23-2[94-16/HD	5.00	43	1.67	43
457	TY/NT-1（2）	3.00	41	5.00	42
458	TY/NT-1（3）	2.33	48	3.67	46
459	tong/dahuapi	5.67	44	4.33	43

Table S3. (Cont.)

S. No.	Lines	2018JP–natural		2018DT–natural	
		DI	FT	DI	FT
460	88-48/sb-f-P1（2）	4.33	40	1.00	42
461	huaidou6hao×kexin3hao	4.33	45	3.00	42
462	IIfu×yu	3.00	50	4.33	49
463	you96-3	4.33	41	5.00	41
464	you4/5545（3）	5.00	43	6.33	45
465	Y-10-05	1.67	41	3.67	45
466	he95-1×peila	5.00	40	1.67	42
467	he95-1	3.00	52	5.00	54
468	huaidou6hao×liaodou11	9.00	53	5.00	54
469	meng9024×peila	3.00	42	6.33	48
470	youbian30×yudou22（1）	2.33	43	2.33	45
471	Y-10-02	4.33	44	2.33	49
472	yudou22×peila	5.00	44	1.00	44
473	0	5.67	49	3.00	48
474	youbian30×yudou22（2）	4.33	48	1.67	50
475	tongdou8hao	5.00	37	1.67	36
476	JN03136×tai292	5.00	37	1.00	40

Table S4. Spearman correlation coefficient of flowering time and BLP resistance in the soybean association panel under the natural morbidity condition

Trait	Environment	BLP resistance		Flowering time	
		**2018JP–natural**	**2018DT–natural**	**2018JP–natural**	**2018DT–natural**
BLP resistance	2018JP–natural	1			
	2018DT–natural	0.01	1		
Flowering time	2018JP–natural	-0.29*	-0.04	1	
	2018DT–natural	-0.28*	-0.04	0.87*	1

*significant at $P < 0.05$

Table S5. Information on marker numbers and map lengths of different chromosome in the RIL population of soybean.

Chromosome	SNP number	Bin number	Linkage distance (cM)	Density (SNP/cM)
Gm01	4,377	117	122.07	1.04
Gm02	2,938	144	158.65	1.1
Gm03	4,258	117	94.79	0.81
Gm04	2,067	120	127.1	1.06
Gm05	1,691	109	99.34	0.91
Gm06	4,845	158	166.56	1.05
Gm07	2,149	135	124.05	0.92
Gm08	4,256	185	187.33	1.01
Gm09	3,415	131	127.99	0.98
Gm10	4,407	137	133.31	0.97
Gm11	1,107	123	143.2	1.16
Gm12	1,682	831	3.29	1.24
Gm13	2,899	158	178.51	1.13
Gm14	1,207	106	116.03	1.09
Gm15	5,560	126	123.11	0.98
Gm16	2,207	113	116.36	1.03
Gm17	2,813	119	135.9	1.14

Table S5. (Cont.)

Chromosome	SNP number	Bin number	Linkage distance (cM)	Density (SNP/cM)
Gm18	7,704	165	130.35	0.79
Gm19	5,374	114	133.88	1.17
Gm20	1,721	140	108.42	0.77
Total	66,677	2,600	2,630.24	1.01

Table S6. QTNs associated with BLP resistance identified by GWAS in the soybean association panel under the artificial inoculation with Xag C5 strain

S. No.	SNP ID	Chromosome	Position	$-\log_{10}P$	R^2(%)	Environment
1	Gm05_7667820	5	7667820	4.06	3.30	2016JP–spray
2	Gm05_7668047	5	7668047	4.06	3.30	2016JP–spray
3	Gm05_7993347	5	7993347	4.42	3.65	2016JP–spray
4	Gm05_8004022	5	8004022	4.96	4.16	2016JP–spray
5	Gm05_8032322	5	8032322	4.64	3.85	2016JP–spray
6	Gm05_8032346	5	8032346	4.64	3.85	2016JP–spray
7	Gm05_8091323	5	8091323	5.66	4.85	2016JP–spray
8	Gm05_8107119	5	8107119	5.66	4.85	2016JP–spray
9	Gm05_8107147	5	8107147	5.66	4.85	2016JP–spray
10	Gm05_8149446	5	8149446	4.94	4.14	2016JP–spray
11	Gm05_8149459	5	8149459	4.65	3.86	2016JP–spray
12	Gm05_8162286	5	8162286	5.52	4.71	2016JP–spray
13	Gm05_8169971	5	8169971	5.52	4.71	2016JP–spray
14	Gm05_8174502	5	8174502	5.52	4.71	2016JP–spray
15	Gm05_8191002	5	8191002	5.25	4.44	2016JP–spray
16	Gm07_4368039	7	4368039	4.56	3.78	2014JP–spray
17	Gm08_7075530	8	7075530	4.02	3.28	2015JP–spray

Table S6. (Cont.)

S. No.	SNP ID	Chromosome	Position	$-log_{10}P$	R^2(%)	Environment
18	Gm08_7075839	8	7075839	4.02	3.28	2015JP–spray
19	Gm08_7075872	8	7075872	4.02	3.28	2015JP–spray
20	Gm08_7075885	8	7075885	4.02	3.28	2015JP–spray
21	Gm09_36500790	9	36500790	4.12	3.37	2015JP–spray
22	Gm09_36501019	9	36501019	4.65	3.87	2014JP–spray
				4.24	3.48	2015JP–spray
23	Gm15_13703612	15	13703612	4.01	3.26	2016JP–spray
24	Gm15_13726816	15	13726816	4.07	3.31	2016JP–spray
25	Gm17_5628119	17	5628119	4.64	3.86	2016JP–spray
26	Gm17_5628133	17	5628133	4.64	3.86	2016JP–spray
27	Gm17_5658566	17	5658566	4.87	4.08	2016JP–spray
28	Gm17_7491104	17	7491104	5.76	4.94	2016JP–spray
29	Gm17_7603802	17	7603802	4.14	3.39	2014JP–spray
				4.99	4.20	2015JP–spray
				6.87	6.04	2016JP–spray
30	Gm17_7603992	17	7603992	4.14	3.39	2014JP–spray
				4.99	4.20	2015JP–spray
				6.87	6.04	2016JP–spray

Table S6. (Cont.)

S. No.	SNP ID	Chromosome	Position	-$log_{10}P$	R^2(%)	Environment
31	Gm17_7604008	17	7604008	5.04	4.24	2016JP–spray
32	Gm17_7712768	17	7712768	5.13	4.35	2015JP–spray
				6.77	5.94	2016JP–spray
33	Gm17_7721556	17	7721556	4.95	4.17	2015JP–spray
				6.46	5.64	2016JP–spray
34	Gm17_7736150	17	7736150	4.33	3.58	2015JP–spray
				5.19	4.39	2016JP–spray
35	Gm17_7754016	17	7754016	4.05	3.30	2014JP–spray
				5.11	4.32	2015JP–spray
				6.66	5.83	2016JP–spray
36	Gm17_7754048	17	7754048	4.05	3.30	2014JP–spray
				5.11	4.32	2015JP–spray
				6.66	5.83	2016JP–spray
37	Gm19_48684769	19	48684769	4.06	3.30	2016JP–spray
38	Gm20_1033092	20	1033092	4.01	3.26	2015JP–spray

Table S7. QTNs associated with BLP resistance identified by GWAS in soybean association panel under natural morbidity condition

S. No.	SNP ID	Chromosome	Position	-log$_{10}P$	R^2(%)	Environment
1	Gm04_7895886	4	7895886	4.08	3.30	2018DT–natural
2	Gm05_7667820	5	7667820	4.21	3.42	2018DT–natural
3	Gm05_7668047	5	7668047	4.21	3.42	2018DT–natural
4	Gm05_38337589	5	38337589	4.32	3.53	2018DT–natural
5	Gm06_2745725	6	2745725	4.07	3.29	2018DT–natural
6	Gm09_41273199	9	41273199	4.59	3.78	2018DT–natural
7	Gm09_41273321	9	41273321	4.31	3.52	2018DT–natural
8	Gm09_41378897	9	41378897	4.33	3.53	2018DT–natural
9	Gm13_11075530	13	11075530	4.13	3.31	2018JP–natural
10	Gm16_29211869	16	29211869	5.19	4.36	2018DT–natural
11	Gm16_29315504	16	29315504	4.86	4.04	2018DT–natural
12	Gm17_5628119	17	5628119	4.45	3.65	2018DT–natural
13	Gm17_5628133	17	5628133	4.45	3.65	2018DT–natural
14	Gm17_5939123	17	5939123	4.08	3.30	2018DT–natural
15	Gm17_6138216	17	6138216	4.23	3.44	2018DT–natural
16	Gm17_6138528	17	6138528	4.23	3.44	2018DT–natural
17	Gm17_7590819	17	7590819	5.85	5.00	2018DT–natural

Table S7. (Cont.)

S. No.	SNP ID	Chromosome	Position	-log$_{10}$*P*	R^2(%)	Environment
18	Gm17_7603802	17	7603802	8.74	7.88	2018DT–natural
19	Gm17_7604008	17	7604008	6.76	5.89	2018DT–natural
20	Gm17_7712768	17	7712768	4.13	3.31	2018JP–natural
				8.17	7.30	2018DT–natural
21	Gm17_7721556	17	7721556	8.32	7.45	2018DT–natural
22	Gm17_7736150	17	7736150	6.68	5.81	2018DT–natural
23	Gm17_7754016	17	7754016	8.14	7.26	2018DT–natural
24	Gm17_7754048	17	7754048	8.14	7.26	2018DT–natural
25	Gm17_8782918	17	8782918	4.22	3.39	2018JP–natural
26	Gm17_9327571	17	9327571	5.71	4.86	2018DT–natural
27	Gm17_9366402	17	9366402	4.04	3.26	2018DT–natural
28	Gm17_9376310	17	9376310	4.04	3.26	2018DT–natural
29	Gm18_58342389	18	58342389	4.64	3.83	2018DT–natural
30	Gm20_186175	20	186175	4.28	3.49	2018DT–natural
31	Gm20_190799	20	190799	4.25	3.46	2018DT–natural
32	Gm20_41883151	20	41883151	4.80	3.94	2018JP–natural
33	Gm20_41883183	20	41883183	4.80	3.94	2018JP–natural
34	Gm20_41894159	20	41894159	4.80	3.94	2018JP–natural

Table S8. Annotated genes in the genomic region 3 on chromosome 17

**Wm82.a1.v1**	**Gene Start (bp)**	**Gene End (bp)**	**Wm82.a2.1**	**Gene Name**
Glyma17g09160	6774921	6778817	*Glyma.17g084100*	N.A.
Glyma17g09170	6784848	6787877	*Glyma.17g084300*	Calcium sensing receptor, chloroplastic-like(*CAS*)
Glyma17g09180	6790554	6792378	*Glyma.17g084400*	Pentatricopeptide repeat-containing protein *At3G48250*, chloroplastic
Glyma17g09190	6794828	6795548	*Glyma.17g084500*	N.A.
Glyma17g09200	6795624	6796225	*Glyma.17g084600*	N.A.
Glyma17g09210	6797766	6802424	*Glyma.17g084700*	Neighbor of BRCA1 gene 1 protein (*NBR1*) homolog
Glyma17g09220	6806438	6807175	*Glyma.17g084800*	N.A.
Glyma17g09230	6808546	6808724	N.A.	N.A.
Glyma17g09240	6815890	6816825	*Glyma.17g084900*	N.A.
Glyma17g09250	6826138	6828802	*Glyma.17g085000*	L-type lectin-domain containing receptor kinase S.1-like(LOC100816035)
Glyma17g09260	6829022	6832521	*Glyma.17g085100*	Ferric reduction oxidase 8, mitochondrial-like
Glyma17g09270	6841390	6846524	*Glyma.17g085300*	Dead-box ATP-dependent RNA helicase 30-like
Glyma17g09280	6847806	6849068	*Glyma.17g085400*	60S ribosomal protein L27a (LOC100305936)
Glyma17g09290	6856298	6861173	*Glyma.17g085500*	Copper methylamine oxidase-like
Glyma17g09300	6863875	6866024	*Glyma.17g085500*	Copper methylamine oxidase-like

Table S8. (Cont.)

Wm82.a1.v1	Gene Start (bp)	Gene End (bp)	Wm82.a2.1	Gene Name
Glyma17g09310	6873898	6875459	*Glyma.17g085600*	N.A.
Glyma17g09320	6890143	6893993	*Glyma.17g085700*	Histone deacetylase (*HDA16*)
Glyma17g09330	6894658	6896034	*Glyma.17g085800*	Probable polyamine amiNopropyl transferase
Glyma17g09340	6896271	6896386	*Glyma.17g085900*	N.A.
Glyma17g09350	6898684	6901436	*Glyma.17g086000*	Dentin sialophospho protein
Glyma17g09360	6901892	6902669	*Glyma.17g086100*	N.A.
Glyma17g09370	6911329	6911582	*Glyma.17g086200*	N.A.
Glyma17g09380	6927230	6928157	*Glyma.17g086300*	Lateral organ boundaries (*LOB*) domain-containing protein 25
Glyma17g09390	6936149	6937109	*Glyma.17g086400*	Oleosin H2
Glyma17g09400	6937495	6939148	*Glyma.17g086500*	Tryptophan amiNotransferase-related protein 2
Glyma17g09410	6954078	6960639	*Glyma.17g086600*	FKBP12-interacting protein of 37 kDa
Glyma17g09420	6968155	6971324	*Glyma.17g086800*	Short-chain dehydrogenase TIC 32 B, chloroplastic
Glyma17g09430	6969759	6978455	N.A.	N.A.
Glyma17g09440	6996816	7001067	*Glyma.17G087000*	Leucine-rich repeat receptor-like serine/threonine-protein kinase *RGI4*
Glyma17g09450	7009991	7013398	*Glyma.17G087100*	N.A.

Table S8. (Cont.)

Wm82.a1.v1	Gene Start (bp)	Gene End (bp)	Wm82.a2.1	Gene Name
Glyma17g09460	7023131	7024972	*Glyma.17G087200*	Plant UBX domain-containing protein 10
Glyma17g09470	7026742	7029916	*Glyma.17G087300*	Probable apyrase 7
Glyma17g09480	7029797	7030508	N.A.	N.A.
Glyma17g09490	7031903	7034163	*Glyma.17G087400*	CRAL-TRIO domain-containing protein C3H8.02
Glyma17g09500	7036148	7037323	*Glyma.17G087500*	SOUL heme-binding domain-containing protein
Glyma17g09510	7041808	7050632	*Glyma.17G087600*	Protein modifier OF *SNC1-1*
Glyma17g09520	7060809	7061137	N.A.	N.A.
Glyma17g09530	7066818	7069403	*Glyma.17G087700*	LRR receptor-like serine/threonine-protein kinase *GSO1*
Glyma17g09540	7075568	7076836	*Glyma.17G087900*	Protein Microrchidia 6
Glyma17g09550	7077316	7088589	*Glyma.17G087900*	Protein Microrchidia 6
Glyma17g09570	7097299	7101389	*Glyma.17G088000*	Cysteine-rich receptor-like protein kinase
Glyma17g09580	7104637	7107799	*Glyma.17G088100*	N.A.
Glyma17g09590	7108382	7114066	*Glyma.17G088200*	U3 small nucleolar ribonucleoprotein protein *MPP10*
Glyma17g09600	7112777	7113981	N.A.	N.A.
Glyma17g09610	7116739	7119805	*Glyma.17G088300*	N.A.
Glyma17g09620	7123333	7127569	*Glyma.17G088400*	N.A.

Table S8. (Cont.)

**Wm82.a1.v1**	**Gene Start (bp)**	**Gene End (bp)**	**Wm82.a2.1**	**Gene Name**
Glyma17g09630	7132241	7136384	*Glyma.17G088500*	Triose phosphate/phosphate translocator, Non-green plastid, chloroplastic
Glyma17g09640	7141687	7143538	*Glyma.17G088600*	Transcription factor *MYB98*
Glyma17g09650	7145595	7150076	*Glyma.17G088700*	*GDSL* esterase/lipase *At5G62930*
Glyma17g09660	7154443	7159511	*Glyma.17G088800*	N.A.
Glyma17g09670	7167136	7169104	*Glyma.17G088900*	N.A.
Glyma17g09680	7169985	7171100	*Glyma.17G089000*	Phosphate kinase (*ITPK2*)
Glyma17g09690	7171843	7185888	*Glyma.17G089100*	Transducin beta-like protein 3
Glyma17g09700	7191302	7202806	*Glyma.17G089200*	Pantothenate kinase 2
Glyma17g09710	7204307	7205487	*Glyma.17G089300*	Dof zinc finger protein *DOF5.6*
Glyma17g09720	7219274	7223407	*Glyma.17G089400*	Probable inactive poly [ADP-ribose] polymerase *SRO5*
Glyma17g09730	7235293	7235563	*Glyma.17G089500*	N.A.
Glyma17g09740	7241079	7245959	*Glyma.17G089600*	1-deoxy-D-xylulose 5-phosphate reductoisomerase, chloroplastic
Glyma17g09750	7251119	7257374	*Glyma.17G089700*	Peroxisomal membrane protein PEX14-like
Glyma17g09760	7262336	7261569	N.A.	N.A.
Glyma17g09770	7269506	7272294	*Glyma.17G090000*	Serine/threonine-protein kinase HT1-like

Table S8. (Cont.)

Wm82.a1.v1	Gene Start (bp)	Gene End (bp)	Wm82.a2.1	Gene Name
Glyma17g09780	7285718	7286797	*Glyma.17G090100*	CASP-like protein 4A3
Glyma17g09790	7293153	7299668	*Glyma.17G090200*	E3 ubiquitin-protein ligase SIS3-like
Glyma17g09801	7305555	7307649	*Glyma.17G090400*	N.A.
Glyma17g09810	7317349	7318511	*Glyma.17G090500*	ACHAETE-SCUTE TRANSCRIPTION FACTOR-RELATED
Glyma17g09820	7331150	7331902	N.A.	N.A.
Glyma17g09830	7336111	7341153	*Glyma.17G090600*	Serine/threonine-protein kinase STY13
Glyma17g09840	7343474	7345735	*Glyma.17G090800*	Sugar efflux transporter SWEET43
Glyma17g09850	7348179	7350515	*Glyma.17G090900*	U-box domain-containing protein 19
Glyma17g09860	7359895	7364584	*Glyma.17G091000*	N.A.
Glyma17g09870	7374757	7376961	*Glyma.17G091100*	Spermidine synthase 2
Glyma17g09880	7377832	7380057	*Glyma.17G091200*	N.A.
Glyma17g09890	7382613	7384051	N.A.	N.A.
Glyma17g09900	7386583	7387764	*Glyma.17G091300*	N.A.
Glyma17g09910	7390673	7391424	*Glyma.17G091400*	N.A.
Glyma17g09920	7392680	7397877	*Glyma.17G091500*	RNA-dependent RNA polymerase 2
Glyma17g09930	7398901	7400181	*Glyma.17G091600*	RING-H2 finger protein ATL47

Table S8. (Cont.)

Wm82.a1.v1	Gene Start (bp)	Gene End (bp)	Wm82.a2.1	Gene Name
Glyma17g09940	7406532	7413534	*Glyma.17G091700*	Ubiquitin-conjugating enzyme E2 4
Glyma17g09950	7418849	7420458	*Glyma.17G091800*	Walls are thin 1 (*WAT1*) -related protein *At5G40230*
Glyma17g09960	7423322	7425402	*Glyma.17G091900*	Uncharacterized LOC100790520
Glyma17g09970	7425703	7431101	*Glyma.17G092000*	Protease Do-like 9 (*DEGP9*)
Glyma17g09980	7434129	7437803	*Glyma.17G092100*	Rho-related GTPase
Glyma17g09990	7440289	7445234	*Glyma.17G092200*	N.A.
Glyma17g10000	7457627	7463878	*Glyma.17G092300*	Nucleobase-ascorbate transporter 6 (*NAT6*)
Glyma17g10010	7465205	7469158	*Glyma.17G092400*	Spermidine synthase-like (*SPDS*)
Glyma17g10020	7485423	7487055	*Glyma.17G092500*	N.A.
Glyma17g10030	7489954	7494188	*Glyma.17G092600*	LIM domain-containing protein WLIM1
Glyma17g10040	7498741	7501948	*Glyma.17G092700*	Calmodulin-binding protein 60 A
Glyma17g10050	7524028	7526123	*Glyma.17G092800*	Gibberellin-regulated protein 26 (*GASA26*)
Glyma17g10060	7528212	7534901	*Glyma.17G092900*	Alpha-manNosidase I MNS5
Glyma17g10070	7536003	7538057	*Glyma.17G093000*	Putative DNA glycosylase *At3G47830*
Glyma17g10080	7542027	7542776	*Glyma.17G093200*	N.A.
Glyma17g10090	7548570	7553330	*Glyma.17G093300*	N.A.
Glyma17g10100	7558655	7559975	*Glyma.17G093400*	Protein JINGUBANG

Table S8. (Cont.)

Wm82.a1.v1	Gene Start (bp)	Gene End (bp)	Wm82.a2.1	Gene Name
Glyma17g10110	7569770	7570478	*Glyma.17G093500*	N.A.
Glyma17g10120	7572287	7575549	N.A.	N.A.
Glyma17g10130	7575891	7580101	*Glyma.17G093600*	*E2F* transcription factor-like E2FE
Glyma17g10140	7582198	7583233	*Glyma.17G093700*	N.A.
Glyma17g10150	7586556	7590008	*Glyma.17G093800*	N.A.
Glyma17g10160	7590737	7591233	N.A.	N.A.
Glyma17g10170	7596412	7598837	*Glyma.17G093900*	Two-component response regulator ARR5-like
Glyma17g10180	7599870	7603103	N.A.	N.A.
Glyma17g10190	7611489	7613101	*Glyma.17G094000*	LisH domain-containing protein C1711.05
Glyma17g10200	7621522	7624014	*Glyma.17G094100*	Microtubule-associated protein TORTIFOLIA1-like
Glyma17g10210	7627559	7628516	N.A.	N.A.
Glyma17g10220	7631411	7631670	N.A.	N.A.
Glyma17g10230	7634749	7637391	*Glyma.17G094200*	N.A.
Glyma17g10240	7645035	7653573	*Glyma.17G094300*	Pentatricopeptide repeat-containing protein *At1G74850*, chloroplastic-like
Glyma17g10250	7663874	7665962	*Glyma.17G094400*	*MYB* transcription factor *MYB173*
Glyma17g10260	7672659	7673593	*Glyma.17G094600*	N.A.

Table S8. (Cont.)

**Wm82.a1.v1**	**Gene Start (bp)**	**Gene End (bp)**	**Wm82.a2.1**	**Gene Name**
Glyma17g10270	7674424	7678675	*Glyma.17G094700*	Serine/threonine-protein kinase AtPK2/AtPK19-like
Glyma17g10280	7680661	7702277	*Glyma.17G094800*	Transportin *MOS14*
Glyma17g10290	7707794	7711352	*Glyma.17G095000*	Transcription factor bHLH79-like
Glyma17g10300	7712542	7713696	*Glyma.17G095100*	N.A.
Glyma17g10310	7727276	7727384	N.A.	N.A.
Glyma17g10320	7727800	7729329	*Glyma.17G095200*	N.V.
Glyma17g10330	7732414	7736034	*Glyma.17G095300*	Hydroxyproline O-galactosyltransferase GALT6
Glyma17g10340	7737006	7737797	*Glyma.17G095400*	60S ribosome subunit biogenesis protein NIP7 homolog
Glyma17g10350	7741539	7743299	*Glyma.17G095500*	AAA-ATPase *At3G28580*
Glyma17g10360	7744740	7753192	N.A.	N.A.

Table S9. Primer sequences used in the present study.

Gene	Function	Primer Name[a]	Primer Sequence (5′–3′)
Glyma.17G090100	CASP-like protein 4A3	Gm17G090100-qPCR-F1	CGGTGGTGCCGAAGAGAGAA
		**Gm17G090100-qPCR-F2**	**GGTGGTGCCGAAGAGAGAAA**
		Gm17G090100-qPCR-R1	CGCTCCAACCACGAGTTTTG
		**Gm17G090100-qPCR-R2**	**AATCACCGCTCCAACCACGA**
Glyma.17G090200	E3 ubiquitin-protein ligase	**Gm17G090200-qPCR-F1**	**TGGTTCAGCAGTGCCAAGAAC**
	SIS3 isoform X1	Gm17G090200-qPCR-F2	AACTGTTTGCCTGAAGTGGG
		**Gm17G090200-qPCR-R1**	**CCAGAGACATGCAAGCAATG**
		Gm17G090200-qPCR-R2	AACCACTTCCCCAGAGACAT
Glyma.17G090400	serine/threonine kinase ;	Gm17G090400-qPCR-F1	GTGAGCTTTTCGACCGGATC
	Calcium-dependent protein	**Gm17G090400-qPCR-F2**	**AGCTTTTCGACCGGATCATC**
	kinase 15	**Gm17G090400-qPCR-R1**	**TCCCATGGTGTGGCAATCGTG**
		Gm17G090400-qPCR-R2	ACTCCCATGGTGTGGCAATCG
Glyma.17G086300	Lateral organ boundaries (LOB) domain-containing protein 25	**Gm17G086300-qPCR-F1**	**TTACACTGACATCACTCCAG**
		Gm17G086300-qPCR-F2	ACACTGACATCACTCCAGCA
		Gm17G086300-qPCR-R1	CACTAAACTGCCTCTGGGGA
		**Gm17G086300-qPCR-R2**	**ATCTTGCACTAAACTGCCTC**
Glyma.05G040500	Lateral organ boundaries (LOB) domain-containing protein 25	Gm05G040500-qPCR-F1	GAATCTAGAACTGCTTACAG
		**Gm05G040500-qPCR-F2**	**CTTACAGAAGAACTTGTGGG**
		**Gm05G040500-qPCR-R1**	**CCCTGCACTAGAAAGATTGG**

Table S9. (Cont.)

Gene	Function	Primer Name[a]	Primer Sequence (5′–3′)
		Gm05G040500-qPCR-R2	TGTAAGAGCTGGATGATGCC

[a]The best primer pair for qPCR of each gene.

Table S10. The optimized qPCR conditions for the five candidate BLP resistance genes in soybean.

Gene Name	Primer Pair[a]	Amplicon Length (bp)	Optimal Tm (°C)[b]	Optimal Primer Concentration (mM)	cDNA Dilution	R^2	E (%)[c]
Glyma.17G090100	Gm17G090100-qPCR-F1/ Gm17G090100-qPCR-R1	123	62.7	350	1/10 - 1/80	0.8292	132.31
	Gm17G090100-qPCR-F1/ Gm17G090100-qPCR-R2	129	62.7	350	1/10 - 1/40	0.9766	137.41
	Gm17G090100-qPCR-F2/ Gm17G090100-qPCR-R1	122	62.7	400	1/10 - 1/160	0.9639	105.50
	**Gm17G090100-qPCR-F2/ Gm17G090100-qPCR-R2**	**128**	**62.7**	**350**	**1/10 - 1/80**	**0.9885**	**103.96**
Glyma.17G090200	**Gm17G090200-qPCR-F1/ Gm17G090200-qPCR-R1**	**112**	**62.7**	**400**	**1/20 - 1/160**	**0.9996**	**100.74**
	Gm17G090200-qPCR-F1/ Gm17G090200-qPCR-R2	122	62.7	400	1/10 - 1/40	0.9998	113.3
	Gm17G090200-qPCR-F2/ Gm17G090200-qPCR-R1	94	62.7	350	1/40 - 1/160	0.9958	103.34
	Gm17G090200-qPCR-F2/ Gm17G090200-qPCR-R2	104	62.7	400	1/10 - 1/160	0.9707	103.83

Table S10. (Cont.)

Gene Name	Primer Pair[a]	Amplicon Length (bp)	Optimal Tm (°C)[b]	Optimal Primer Concentration (mM)	cDNA Dilution	R^2	E (%)[c]
Glyma.17G090400	Gm17G090400-qPCR-F1/ Gm17G090400-qPCR-R1	107	62.7	350	1/40 - 1/160	0.9057	112.14
	Gm17G090400-qPCR-F1/ Gm17G090400-qPCR-R2	109	62.7	400	1/10 - 1/40	0.9985	109.6
	**Gm17G090400-qPCR-F2/ Gm17G090400-qPCR-R1**	**104**	**62.7**	**350**	**1/10 - 1/80**	**0.9966**	**103.24**
	Gm17G090400-qPCR-F2/ Gm17G090400-qPCR-R2	106	62.7	350	1/10 - 1/160	0.9713	104.43
Glyma.17G086300	Gm17G086300-qPCR-F1/ Gm17G086300-qPCR-R1	112	60.6	300	1/10 - 1/80	94.87	112.8
	**Gm17G086300-qPCR-F1/ Gm17G086300-qPCR-R2**	**120**	**60.6**	**350**	**1/10 - 1/40**	**99.9**	**107.3**
	Gm17G086300-qPCR-F2/ Gm17G086300-qPCR-R1	110	60.6	250	1/10 - 1/80	63.53	152.5
	Gm17G086300-qPCR-F2/ Gm17G086300-qPCR-R2	118	60.6	400	1/10 - 1/80	56.89	179.09
Glyma.05G040500	Gm05G040500-qPCR-F1/ Gm05G040500-qPCR-R1	88	-	-	-	-	-
	Gm05G040500-qPCR-F1/ Gm05G040500-qPCR-R2	105	-	-	-	-	-
	**Gm05G040500-qPCR-F2/ Gm05G040500-qPCR-R1**	**75**	**60.6**	**400**	**2/5 - 1/10**	**99.79**	**100.46**

Table S10. (Cont.)

Gene Name	Primer Pair[a]	Amplicon Length (bp)	Optimal Tm (°C)[b]	Optimal Primer Concentration (mM)	cDNA Dilution	R^2	E (%)[c]
	Gm05G040500-qPCR-F2/ Gm05G040500-qPCR-R2	92	60.6	400	2/5 - 1/10	99.59	108.77

[a]The best primer pair for each gene was in bold. [b]Tm, annealing temperature. [c]E (%), efficiency.

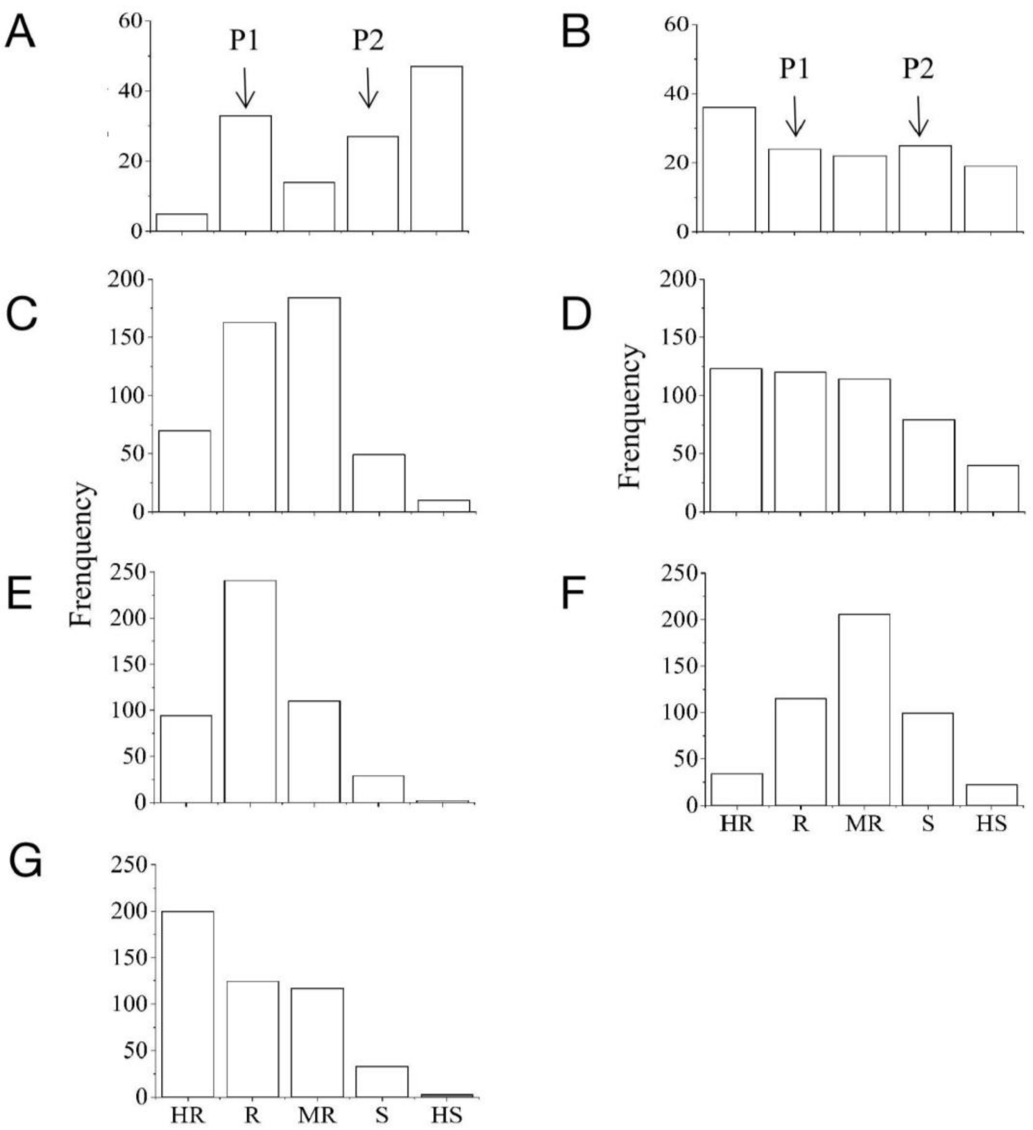

Figure S1. Frequency distribution of phenotypic traits of a soybean RIL population and an association panel of soybean breeding lines resistance to BLP disease in soybean. (A-B) the frequency distribution of phenotypic traits of the RIL population under the artificial inoculation condition in 2014 and 2015, respectively, and the disease resistance levels of the parents Meng8206 (P1) and Tongshan (P2) were denoted by arrows. **(C-E)** the frequency distribution of phenotypic traits of association panel lines under the artificial inoculation in 2014-2016, respectively. **(F-G)** the frequency distribution of phenotypic traits of association panel lines under the natural morbidity condition in 2018 in Jiangpu and Dangtu, respectively. HR, highly resistant; R, resistant; MR, medium resistant; HS, highly susceptible; S,

susceptible.
